# Quenching mechanism in rotaxane mechanophores: insights from acene-based luminophores

**DOI:** 10.1039/d5sc05343a

**Published:** 2025-10-08

**Authors:** Keigo Nonaka, Hayato Sakai, Ryusei Mori, Naoki Shimada, Shunsuke Hatatsu, Taku Hasobe, Yoshimitsu Sagara

**Affiliations:** a Department of Materials Science and Engineering, Institute of Science Tokyo 2-12-1 Ookayama, Meguro-ku Tokyo 152-8550 Japan sagara@mct.isct.ac.jp; b Department of Chemistry, Faculty of Science and Technology, Keio University Yokohama Kanagawa 223-8522 Japan sakai@chem.keio.ac.jp hasobe@chem.keio.ac.jp; c Research Center for Autonomous Systems Materialogy (ASMat), Institute of Integrated Research, Institute of Science Tokyo 4259 Nagatsuta-cho, Midori-ku Yokohama Kanagawa 226-8501 Japan

## Abstract

Rotaxane-based mechanophores that exploit spatial separation between a luminophore and a quencher are attractive due to their high structural design flexibility, enabling high-contrast changes in fluorescence intensity. However, it remains unclear whether their quenching mechanism is predominantly governed by photoinduced electron transfer (PET) or ground-state charge-transfer (CT) complex formation. This study unveils the quenching mechanism using rotaxane mechanophores incorporating π-extended anthracene, tetracene, or pentacene. In toluene, the quenching efficiency decreases with increasing π-conjugation of the fluorophore. Steady-state and transient absorption spectroscopy clarify that the fluorescence quenching of the anthracene-containing rotaxane is primarily due to PET, with a minor contribution from CT complex formation. In contrast, no clear CT complex formation is observed for the tetracene- and pentacene-containing mechanophores. PET moderately quenches the fluorescence for the tetracene-based system, while the low PET efficiency in the pentacene-containing mechanophore results in minimal quenching. Polyurethane elastomer films containing the anthracene-based mechanophore exhibit a significant increase in fluorescence intensity upon mechanical deformation. In contrast, almost no activation is observed for the pentacene-based mechanophore embedded in polyurethane. These findings clarify that PET is the primary quenching mechanism in rotaxane-based mechanochromic mechanophores, offering valuable insights for the future design of supramolecular mechanophores.

## Abstract 2

The quenching mechanism of the rotaxane mechanochromic mechanophore is investigated with a series of π-extended acene groups. Photoinduced electron transfer is found to be primarily responsible for the quenching.

## Graphical abstract

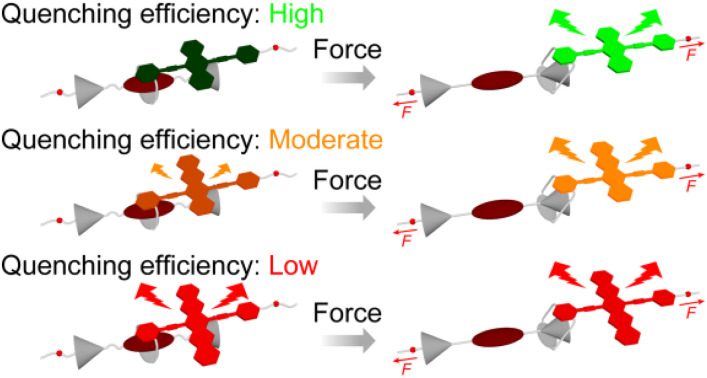

## Introduction

Molecular entities capable of visualizing tiny forces in the pico-to nano-newton range are valuable tools for identifying damaged regions in polymeric materials and elucidating damage propagation mechanisms.^1–6^ Most such “mechanochromic mechanophores” operate through covalent bond scission.^7–20^ Since recovery of cleavage covalent bonds typically requires time or additional energy, these mechanophores often exhibit irreversible behavior, making them suitable for recording force history. In contrast, mechanophores that undergo rapid and reversible photophysical changes enable real-time visualization of mechanical stress.^21–50^ Among these, supramolecular mechanochromic mechanophores—which rely on changes in the spatial arrangement of luminophores and quenchers—have been developed using several structural motifs, including rotaxanes,^39–41,46–50^ loop-like architectures,^24,25,42,44^ and cyclophanes.^36,38,43,45^ Notably, rotaxane-based mechanophores offer significant design versatility. The prototype mechanochromic one comprises a luminescent ring containing a benzothiadiazole-based luminophore and a 1,5-disubstituted naphthalene unit, threaded onto an axle bearing a naphthalene diimide (NDI) quencher and two tetraphenylmethane-based stoppers.^49^ In the absence of applied force, electrostatic interactions between the ring and the quencher maintain their proximity, resulting in fluorescence quenching. When covalently embedded in segmented polyurethane elastomer films, the rotaxane exhibits instantaneous and reversible fluorescence switching upon cyclic stretching, as the ring slides along the axle in response to applied force through the polymer chains. Subsequent studies demonstrated that emission color can be tuned by replacing the luminophore.^47,48^ Moreover, less bulky stoppers permit force-induced dethreading in addition to reversible shuttling, thereby enabling irreversible behavior.^41,46^ Increasing axle length results in delayed fluorescence decay after force removal.^40^ This operating principle has further been employed to modulate fluorescence resonance energy transfer (FRET) efficiency.^39^ More recently, in addition to the conventional Huisgen 1,3-dipolar cycloaddition between an azide and a terminal alkyne groups, amide bond formation between a succinimidyl ester and an amine has emerged as a viable approach for constructing rotaxane mechanophore axles.^50^ The high degree of structural design flexibility inherent to rotaxanes is also exemplified in rotaxane-based mechanophores that rely on the motion of the macrocycle to induce covalent bond scission.^51–56^

Despite these advances, the precise mechanism underlying fluorescence quenching in rotaxane-based mechanochromic mechanophores upon interlocked structure formation remains unclarified. Clarifying this mechanism would provide foundational principles for designing supramolecular mechanophores with enhanced fluorescence modulation. Two plausible quenching pathways have been proposed: (1) photoinduced electron transfer (PET), in which electron acceptors such as NDI or pyromellitic diimide (PMDI) partially or completely accept an electron from an excited donor, thereby forming a charge-transfer (CT) state in the excited state (CT-PET); or (2) formation of a CT complex between the electron donor and the electron acceptor in the ground state (CT-GS) (Fig. S1, SI). Both CT-PET and CT-GS generally display weak or negligible fluorescence, resulting in quenching. We have already found that a rotaxane mechanophore incorporating a π-extended BODIPY red emitter and NDI exhibited poor quenching efficiency in polyurethane elastomers, diminishing emission contrast upon cyclic stretching.^48^ Considering the HOMO–LUMO levels of BODIPY and NDI, both CT-PET and CT-GS are considered inefficient. In this case, the effects of steric hindrance from fluorine substituents should be also considered because the fluorine atoms disturb stable stacking between the luminophore and quencher, further reducing quenching efficiency. Therefore, a series of structurally analogous luminophores is required to systematically investigate the quenching mechanism.

**Fig. 1:**
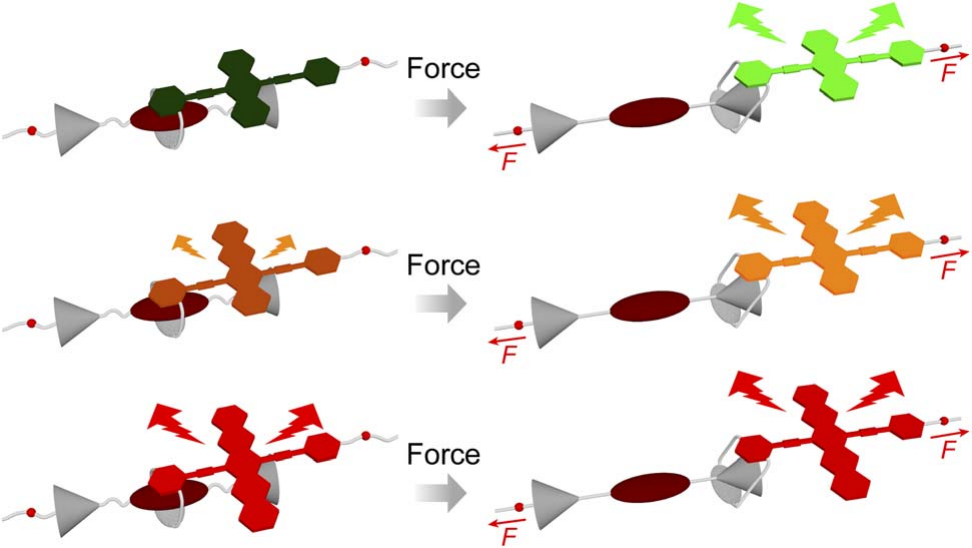
Schematic illustration of rotaxane mechanophores having π-extended acene groups as the emitter before (left) and after (right) force application. Spatial separation occurs between the emitter and quencher (brown) of each rotaxane when force is transduced to each mechanophore. The stoppers (grey) are big enough to prevent force-induced dethreading. Quenching efficiency in the force-free states becomes lower with expanding the π-conjugation.

In this study, we synthesized three supramolecular mechanophores incorporating 9,10-bis(phenylethynyl)anthracene, 5,12-bis(phenylethynyl)tetracene, or 6,13-bis(phenylethynyl)pentacene as an electron-donating unit and PMDI as an electron acceptor within the rotaxane framework (Fig. 1). Extension of the acene π-system modulates both the S_1_ energy and the electron-donating character, enabling systematic tuning of PET efficiencies and CT-GS properties. Photophysical analysis in toluene and polyurethane elastomers revealed that, in all cases, PET was identified as the primary quenching mechanism rather than CT-GS. While tetracene- and pentacene-based rotaxanes showed incomplete quenching, the anthracene-based system exhibited complete fluorescence quenching due to ultrafast PET. These findings provide mechanistic insights for designing supramolecular mechanophores with tunable photophysical properties and efficient luminescence switching.

## Results and discussion

**Fig. 2:**
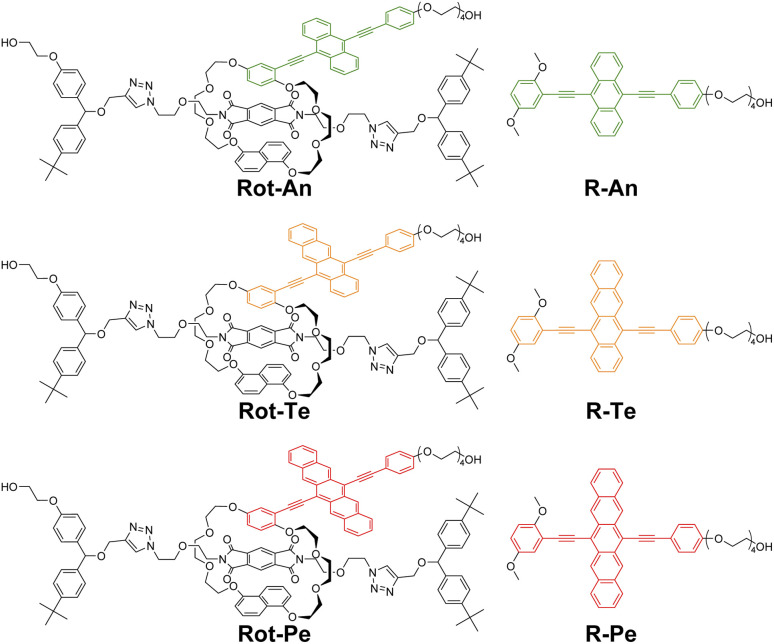
Molecular structures of rotaxanes Rot-An, Rot-Te, and Rot-Pe and reference compounds R-An, R-Te, and R-Pe.

The rotaxane mechanophores incorporating acene fluorophores, along with the reference compounds investigated in this study, are shown in Fig. 2. The rings of Rot-An, Rot-Te, and Rot-Pe incorporate 9,10-bis(phenylethynyl)anthracene, 5,12-bis(phenylethynyl)tetracene, and 6,13-bis(phenylethynyl)pentacene, respectively, as the fluorophore. Although the synthesis, photophysical properties, and mechanoresponsive behavior of Rot-An have been previously reported,^40^ it was re-investigated in this study for direct comparison with the other two rotaxane mechanophores. Given the structural similarity and planarity of these acene-based emitters, the steric hindrance between the emitter and the quencher is expected to be comparable across the three rotaxanes. This structural consistency enables us to examine how the energy gap between the S_1_ state of the fluorophore and the CT-PET correlates with the quenching efficiency in each rotaxane. Simultaneously, the effect of CT-GS formation can also be systematically investigated. Each ring contains both the fluorophore and a 1,5-disubstituted naphthalene unit, which are connected *via* triethylene glycol linkers. Additionally, a flexible tetraethylene glycol chain is appended to each fluorophore to enhance solubility. PMDI (A) is employed as the quencher due to its strong electron-accepting character. Bis(4-*tert*-butylphenyl)methane is used as the stopper, as it has been demonstrated to prevent force-induced dethreading.^40,41^ Two hydroxyl groups are introduced into each rotaxane to allow covalent incorporation into segmented polyurethane elastomers. The compounds R-An, R-Te, and R-Pe serve as acyclic reference molecules for evaluating the photophysical properties of the fluorophores in their monomeric states and for theoretical calculations.

Before syntheses of acene-based rotaxanes, the possibility of PET between acenes and A was evaluated based on the energy levels of the S_1_ states of the acenes and the corresponding excited CT state between acenes and A. The S_1_ energies were estimated from the absorption and fluorescence spectra of reference compounds corresponding to each acene (*vide infra*). The CT state energies in toluene were assessed using the Rehm–Weller equation.^57^ The S_1_ energy levels were estimated to be 2.7 eV (R-An), 2.2 eV (R-Te), and 1.9 eV (R-Pe), while the CT state energies were calculated to be 2.4 eV ((R-An)–(A)), 2.1 eV ((R-Te)–(A)), and 1.9 eV ((R-Pe)–(A)), respectively.^57–62^ These energy relationships suggest that all acenes can form CT states with A in the excited state from S_1_ states. Rot-Te and Rot-Pe were synthesized *via* Huisgen 1,3-dipolar cycloaddition^63,64^ between azide and terminal alkyne groups introduced into the axle precursors. All new compounds were thoroughly characterized by ^1^H NMR, ^13^C NMR, and high-resolution electrospray ionization mass spectrometry (see SI for details). The ^1^H NMR spectra of Rot-Te and Rot-Pe exhibit distinct shifts in the aromatic region upon rotaxane formation, indicating the close proximity of PMDI and 5,12-bis(phenylethynyl)tetracene or 6,13-bis(phenylethynyl)pentacene unit (Fig. S2 and S3, SI).

**Fig. 3:**
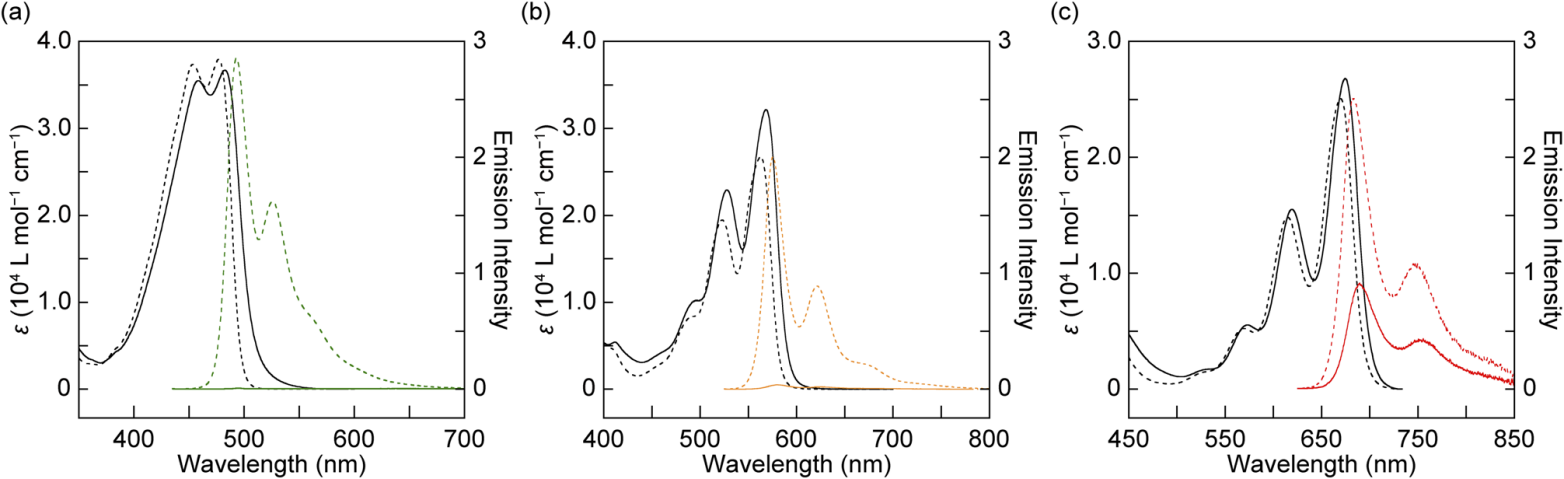
Absorption (black) and photoluminescence (green, orange, and red) spectra of each rotaxane (solid line) and corresponding reference compound (dotted line) in toluene. (a) Rot-An and R-An (*λ*_ex_ = 430 nm), (b) Rot-Te and R-Te (*λ*_ex_ = 520 nm), and (c) Rot-Pe and R-Pe (*λ*_ex_ = 615 nm). Absorption and photoluminescence spectra were recorded with concentrations of 1.0 × 10^−5^ M and 1.0 × 10^−6^ M, respectively.

Steady-state UV-vis absorption and photoluminescence spectra of all rotaxanes and reference compounds were measured in toluene (Fig. 3). The reference compounds R-An, R-Te, and R-Pe exhibit well-resolved vibronic structures in both their absorption and photoluminescence spectra (Fig. 3, dotted lines). As the π-conjugation of the luminophore extends, both the absorption and emission bands are progressively red-shifted. The fluorescence quantum yields (*Φ*) of R-An, R-Te, and R-Pe in toluene are 0.91, 0.73, and 0.37, respectively (Table S1, SI). The slight red shifts of absorption bands upon rotaxane formation are likely due to π-stacked structures constructed among the luminophore, the quencher, and the 1,5-disubstituted naphthalene moiety. These interactions would increase the proportion of planar conformations of the emitters. Rot-An exhibits a distinct absorption tail in the lower energy region, suggesting CT-GS formation between the electron-deficient PMDI quencher and the electron-donating 9,10-bis(phenylethynyl)anthracene. In contrast, no such absorption tail was observed in the spectra of Rot-Te and Rot-Pe, indicating almost no CT-GS formation with the quencher in the two rotaxanes. As shown in Fig. 3a, the fluorescence of Rot-An is completely quenched upon rotaxane formation (*Φ* < 0.01). Rot-Te also exhibits a markedly reduced fluorescence intensity in toluene (*Φ* = 0.01), although its quenching efficiency is slightly lower than that of Rot-An. The lowest quenching efficiency was observed for Rot-Pe, which retains noticeable red fluorescence in toluene (*Φ* = 0.14). These results indicate that quenching efficiency decreases with increasing π-conjugation of the acene unit.

To clarify the extent to which CT-GS formation contributes to the quenching behavior in Rot-An, an absorption titration experiment was conducted using An, which is the ring of Rot-An, and a linear PMDI derivative Ax (Fig. S4, SI). Guest Ax solutions containing the host An were sequentially added to a solution of An, maintaining a constant An concentration throughout the titration. As the concentration of Ax increased, the absorbance attributed to CT-GS formation between An and the PMDI gradually increased (Fig. S5a, SI). Based on this incremental change in absorbance, the population of the CT-GS in Rot-An was estimated to be 18%. This estimate indicates that CT-GS has a small influence on the pronounced fluorescence quenching observed in the Rot-An solution, suggesting that another mechanism, PET, is responsible. It is worth mentioning that the titration experiments were performed in chloroform, a more polar solvent than toluene, because large amounts of Ax cannot dissolve in toluene. Although CT-GS formation is expected to be enhanced in chloroform, the absorption spectra of Rot-An in chloroform and toluene are nearly identical (Fig. S6). However, closer inspection revealed that the absorbance attributed to the CT-GS in chloroform is slightly higher than that in toluene. Therefore, the contribution of CT-GS formation to the fluorescence quenching is considered to be lower in toluene.

**Fig. 4:**
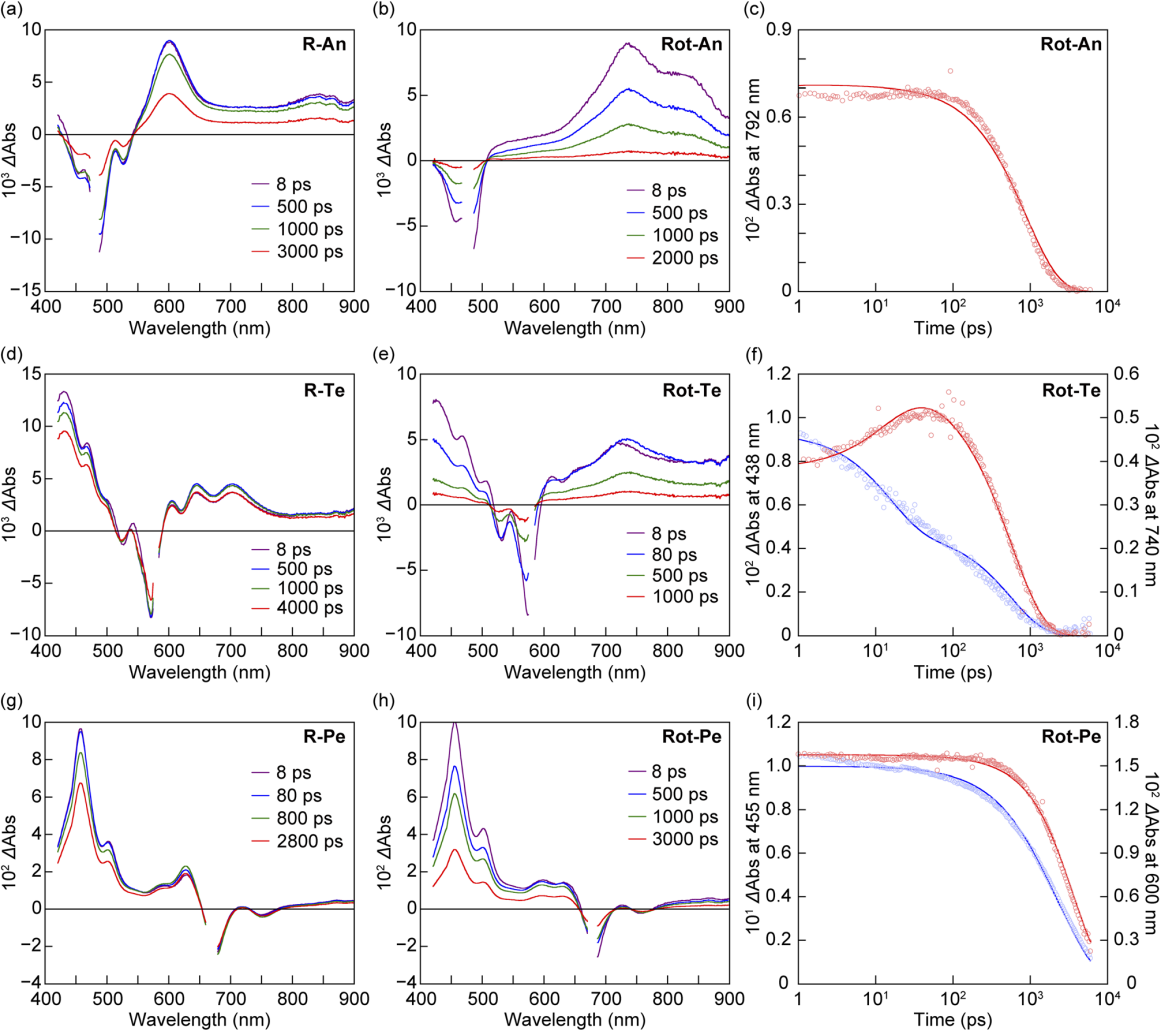
fs-TAS of (a) R-An (*λ*_ex_ = 480 nm), (b) Rot-An (*λ*_ex_ = 480 nm), (d) R-Te (*λ*_ex_ = 580 nm), (e) Rot-Te (*λ*_ex_ = 580 nm), (g) R-Pe (*λ*_ex_ = 670 nm), and (h) Rot-Pe (*λ*_ex_ = 680 nm) in toluene. (c), (f) and (i) transient absorption decay profiles for (c) Rot-An at 792 nm, (f) Rot-Te at 438 nm (blue) and 740 nm (red), and (i) Rot-Pe at 455 nm (blue) and 600 nm (red) in toluene. The blue plots represent the decay of the absorption band predominantly attributed to the S_1_ state, while the red plots represent the decay of the absorption band primarily associated with the CT-PET.

To investigate the quenching process in detail, femtosecond transient absorption spectra (fs-TAS) of three rotaxanes Rot-An, Rot-Te, and Rot-Pe were measured in toluene upon excitation at wavelengths corresponding to their 0–0 bands of acene units (Fig. 4). First, R-An, R-Te, and R-Pe were measured to determine the spectra corresponding to their S_1_ states in toluene (Fig. 4a, d, and g). For all acenes, only a monotonous decay of the spectrum corresponding to the S_1_ was observed immediately after excitation. Next, to examine the quenching process, fs-TAS of the rotaxanes were measured. Upon selective excitation of the fluorophore in Rot-An, no spectrum corresponding to S_1_ was observed; instead, a broad peak with an absorption maximum around 700–800 nm appeared immediately and decayed monotonically within 6000 ps (Fig. 4b). Even from the target analysis of the fs-TAS,^65^ the spectrum of the S_1_ of the fluorophore in Rot-An could not be detected (Fig. S7 and S8, SI), which is attributed to quenching of S_1_ occurring on a timescale faster than the laser pulse duration. Considering spectrum shapes of S_1_ of the fluorophore^58^ and PMDI,^59^ as well as the energy relationship between the S_1_ of the fluorophore and CT in Rot-An, the obtained spectrum is suggested to correspond to the CT-PET. Because the broad peaks are not similar to those of the radical cation of the luminophore^66^ and the radical anion of PMDI,^67^ we assume that the charge separation partially occurs. Based on the analysis results, the rate constant of the PET process (*k*_PET_) was estimated to exceed 2.4 × 10^12^ s^−1^, while the rate constant for charge recombination of the CT-PET (*k*_CR_) was determined to be 1.1 × 10^9^ s^−1^ (Fig. S9a, SI). The fast rate constant *k*_PET_ was not able to correctly determine due to the limitation of time resolution of the instrument. In contrast, in Rot-Te, a characteristic spectrum of the tetracene S_1_ with three absorption maxima in the 400–500 nm range was observed immediately after excitation (Fig. 4e). Around 10 ps, as the S_1_ decayed, broad peaks appeared in the 650–900 nm region. This spectral feature was assigned to the CT-PET due to its similarity in shape to that of the CT-PET observed in Rot-An. Subsequently, a spectrum representing a mixture of S_1_ and CT-PET decayed monotonically, suggesting that S_1_ and CT-PET are in equilibrium. Based on this scheme, target analysis was performed, and spectra corresponding to the S_1_ and CT-PET were obtained (Fig. S8e, SI). The rate constants for PET (*k*_PET_) and its reverse reaction from CT-PET to S_1_ (*k*_BET_) were estimated to be 4.0 × 10^10^ s^−1^ and 2.5 × 10^10^ s^−1^, respectively, and found to be nearly identical. The rate constant for *k*_CR_ was on the order of 10^9^ s^−1^, and the CT-PET yield was estimated to be 55% based on the time-dependent population change (Fig. S7e, SI). In Rot-Pe, the spectrum corresponding to the S_1_ was observed immediately after excitation and was found to decay over time. Notably, when comparing the decay of the absorption band at 455 nm between R-Pe and Rot-Pe, the decay was faster in Rot-Pe. Furthermore, in the fs-TAS of Rot-Pe, the intensity ratio of Δ*A* at 455 nm to that at 600 nm was calculated for the spectra at 8.0 ps and 3000 ps, and the obtained ratios are 7 : 1 and 4 : 1, respectively. These results indicate that the spectrum at 3000 ps includes contributions from species other than the S_1_, such as CT-PET, and that the spectral shape suggests an equilibrium between the respective species. Target analysis of the fs-TAS data for Rot-Pe using a scheme that considers two species resulted in two distinct spectra. One of the spectra showed good agreement with the spectral shape of R-Pe and was therefore assigned to the S_1_, while the other spectrum was assigned to the CT-PET. The rate constants for *k*_PET_, *k*_BET_, and *k*_CR_ were estimated to be on the order of 10^8^ s^−1^ (Fig. S9c, SI). Based on the time-dependent population changes, the CT-PET yield was estimated to be 30%. The estimated CT-PET yields in the rotaxanes showed good agreement with their fluorescence quenching efficiencies (*Vide supra*). A comparison of the *k*_PET_ values among the rotaxanes Rot-An, Rot-Te, and Rot-Pe showed that the rate constant increased as the number of aromatic rings in the acene unit decreased.

Theoretical calculations performed for the model fluorophores and the quencher support these experimental results (Fig. S10 and S11, SI). Specifically, the LUMO levels progressively decrease with extended π-conjugation of acenes. The large energy gap between the LUMO level of 9,10-bis(phenylethynyl)anthracene and that of the PMDI results in a large energy gap between the S_1_ and CT-PET of Rot-An, thereby facilitating efficient PET. In contrast, in Rot-Pe, the LUMO level of 6,13-bis(phenylethynyl)pentacene was calculated to be nearly equivalent to that of the PMDI. The significantly narrower LUMO gap leads to a smaller energy gap between the S_1_ and CT-PET of Rot-Pe, which results in a less efficient PET process and an enhanced rate of reverse reaction from CT-PET to the S_1_. These combined factors collectively suggest a decrease in fluorescence quenching efficiency with increasing aromatic rings.

After characterizing the photophysical properties of the rotaxanes in solutions, Rot-Te and Rot-Pe were individually and covalently embedded into a segmented polyurethane elastomer using the established procedures from our previous studies.^40–50^ Linear segmented polyurethane elastomers are particularly suitable for evaluating the mechanoresponsiveness of supramolecular mechanophores due to their highly stretchable and reversible nature.^68–70^ The rotaxane-containing polyurethanes Rot-Te-PU and Rot-Pe-PU were synthesized *via* a polyaddition reaction involving the corresponding mechanophore, telechelic poly(tetrahydrofuran)diol, 1,4-butanediol, and 4,4′-methylenebis(phenyl isocyanate). Rot-An-PU has been prepared in the previous study^40^ and the same sample was used in the current study for comparison. The concentrations of mechanophores in each polyurethane ranged from 0.13 to 0.22 wt%. The number-average molecular weights (*M*_n_) of Rot-An-PU, Rot-Te-PU, and Rot-Pe-PU were 61 000, 132 000, and 61 000, respectively. Due to the low concentrations of the rotaxanes, the ^1^H NMR spectra of Rot-Te-PU and Rot-Pe-PU showed no discernible peaks corresponding to the embedded rotaxanes (Fig. S12, SI). However, UV-vis absorption and photoluminescence spectra recorded for both polyurethanes in THF exhibited absorption and emission bands attributable to the respective rotaxanes, confirming their successful incorporation into the linear segmented polyurethane chains (Fig. S13 and S14, SI).

Thin films of Rot-Te-PU and Rot-Pe-PU, with thicknesses ranging from 60 to 80 μm, were prepared by solvent casting from THF solutions. Thermogravimetric analysis (TGA) revealed that significant weight loss began at approximately 300 °C (Fig. S15, SI). Differential scanning calorimetry (DSC) curves recorded for all polymers exhibited exothermic peaks around 100 °C during the first cooling cycle and endothermic peaks near 180 °C during the second heating cycle (Fig. S16, SI), corresponding to crystallization and melting of the hard segments of the segmented polyurethanes, respectively. Tensile testing of the pristine films confirmed that the mechanical properties of Rot-Te-PU, and Rot-Pe-PU closely resembled those of the linear segmented polyurethane elastomers prepared in our previous studies (Fig. S17 and Table S4, SI).^40–50^

**Fig. 5:**
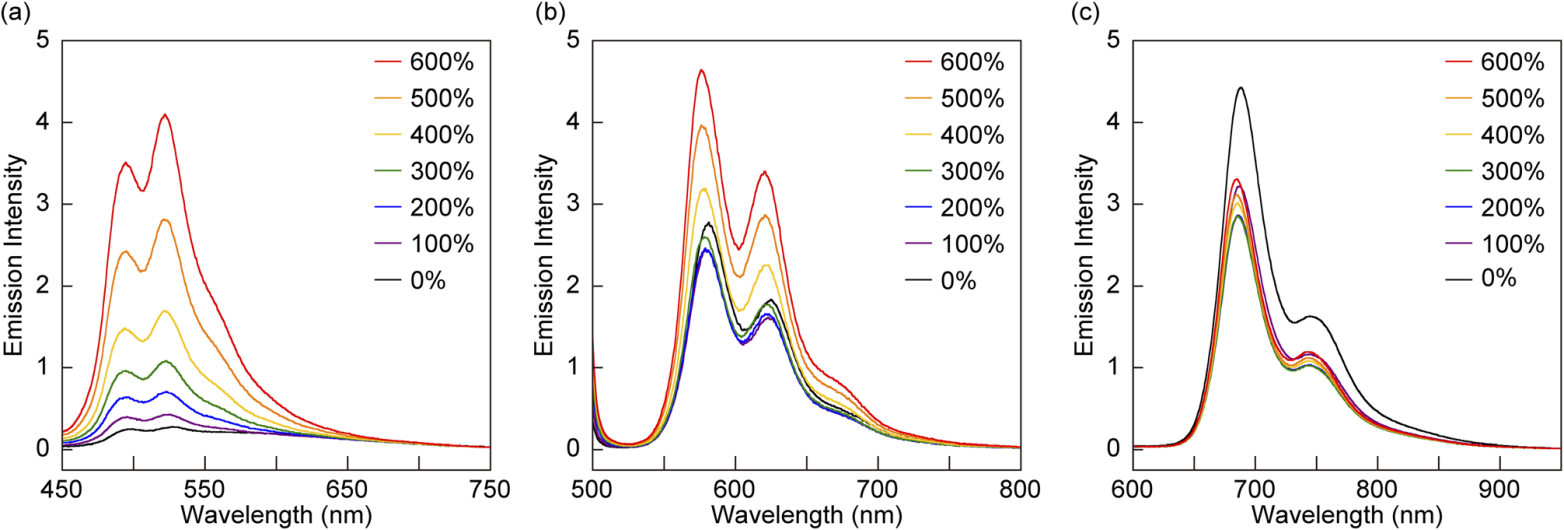
Change of the photoluminescence spectra of (a) Rot-An-PU (*λ*_ex_ = 365 nm), (b) Rot-Te-PU (*λ*_ex_ = 490 nm), and (c) Rot-Pe-PU (*λ*_ex_ = 365 nm) films upon uniaxial deformation to the strains indicated. The spectra were collected under the same experimental conditions.

*In situ* photoluminescence spectroscopy was performed on pristine films of Rot-An-PU, Rot-Te-PU, and Rot-Pe-PU during deformation (Fig. 5). The pristine Rot-An-PU film exhibits faint fluorescence peaks at 499 and 528 nm prior to deformation. This emission is likely due to a small population of ring moieties that are kinetically trapped away from the quencher during solvent casting, resulting in weak green fluorescence. Upon mechanical deformation, the fluorescence intensity of the Rot-An-PU film gradually increases, indicating that Rot-An effectively functions as a supramolecular mechanophore. Although the toluene solution of Rot-Te exhibits only weak orange fluorescence (Fig. 3b), the pristine Rot-Te-PU film displays strong orange emission. Upon deformation, no significant change in fluorescence intensity is observed up to 200% strain. This can be attributed to two opposing effects: an increase in the population of activated Rot-Te and a thinning of the emissive film. Because the illuminated area remains constant while the film stretches, the number of excited Rot-Te molecules per unit area decreases. At strains exceeding 200%, the former effect becomes dominant, resulting in a noticeable increase in fluorescence intensity. This observation suggests that PET-induced quenching remains operative, albeit weakly, for Rot-Te within the polyurethane matrix. The Rot-Pe-PU film also exhibits strong red fluorescence even in the absence of external force, and no significant enhancement in emission was observed upon stretching up to 600% strain. Instead, a gradual decrease in fluorescence intensity occurs, consistent with the dominance of the thinning effect. The emission intensities of the Rot-An-PU and Rot-Te-PU films at 600% strain become 13.7- and 1.64-fold higher relative to the pristine state, respectively, whereas that of the Rot-Pe-PU film at 600% strain shows an emission intensity of 0.76 times that of the initial state. These results indicate that PET contributes minimally to fluorescence quenching in Rot-Pe within the polyurethane films. Overall, it was found that the quenching efficiency is generally lower after incorporation into the polymer matrix, likely due to the presence of a fraction of ring components that are kinetically trapped away from PMDI. These results highlight the importance of achieving effective fluorescence quenching in solution prior to polymer incorporation to obtain a large change in fluorescence intensity upon mechanical activation of rotaxane-based supramolecular mechanophores.

## Conclusions

In conclusion, we successfully elucidated the quenching mechanism of rotaxanes incorporating acene-based emitters. While the quenching mechanisms of previously reported rotaxane mechanophores have remained unclear, our comprehensive investigation—including quantification of charge-transfer (CT) complex proportions and transient absorption spectroscopy—revealed that the efficient photoinduced electron transfer (PET) process is primarily responsible for the complete quenching of green fluorescence in Rot-An, with only a minor contribution from CT complex formation in the ground state. This latter contribution is nearly absent in the other two rotaxane mechanophores, Rot-Te and Rot-Pe. The substantial quenching of orange fluorescence observed for Rot-Te is attributed exclusively to PET. In contrast, the lowest PET efficiency, occurring between the red-emissive 6,13-bis(phenylethynyl)pentacene in Rot-Pe and the quencher, results in minimal fluorescence quenching. These insights provide valuable guidance for the rational design of future supramolecular mechanophores with enhanced responsiveness and versatility.

## Author contributions

K. Nonaka and R. Mori synthesized the rotaxanes. N. Shimada and S. Hatatsu contributed to the synthesis of intermediate compounds. K. Nonaka and R. Mori also performed various spectroscopic measurements. K. Nonaka organized the experimental data. H. Sakai, K. Nonaka, and T. Hasobe conducted transient absorption spectroscopy and performed data analysis. The manuscript was written by K. Nonaka, H. Sakai, T. Hasobe, and Y. Sagara. H. Sakai, T. Hasobe, and Y. Sagara conceived the project and supervised all aspects of the research.

## Conflicts of interest

There are no conflicts to declare.

## Supplementary Material

SC-OLF-D5SC05343A-s001

## Data Availability

The data supporting this article have been included as part of the supplementary information (SI). Supplementary information is available. See DOI: https://doi.org/10.1039/d5sc05343a.
