# Supplementary material for: Quenching mechanism in rotaxane mechanophores: insights from acene-based luminophores
Source: Chem Sci. 2025 Oct 8;16(45):21534–41. doi: 10.1039/d5sc05343a (PMC12523581; doi:10.1039/d5sc05343a)
Supplement: SC-016-D5SC05343A-s001 [file SC-016-D5SC05343A-s001.pdf]

Supplementary Information (SI) for Chemical Science.
This journal is © The Royal Society of Chemistry 2025

Supporting Information

Quenching mechanism in rotaxane mechanophores: insights from acene-based luminophores

Keigo Nonaka, Hayato Sakai,* Ryusei Mori, Naoki Shimada,
Shunsuke Hatatsu, Taku Hasobe,* Yoshimitsu Sagara*

E-mail: sakai@chem.keio.ac.jp; hasobe@chem.keio.ac.jp; sagara@mct.isct.ac.jp

Table of Contents

Experimental Section ········· **S2**
Synthesis of Rotaxanes and Reference Compounds ········· **S4**
Synthesis of Polyurethanes ········· **S11**
Preparation of Polyurethane Films ········· **S12**
Qualitative Jablonski Diagrams ········· **S12**
Method for Estimating the Energy of the CT State ········· **S12**
^{1}H NMR Spectra of Rotaxanes ········· **S13**
Quantum Yields and Fluorescence Lifetime of References and Rotaxanes in Toluene ········· **S15**
Titration Experiments for Estimating the Percentage of CT Complex Formation for Rot-An ········· **S16**
Comparison of the Absorption Spectra of Rot-An in Chloroform and Toluene ········· **S19**
Transient Absorption Spectroscopy ········· **S19**
Theoretical Calculations ········· **S21**
^{1}H NMR Spectra of Polyurethanes ········· **S23**
Absorption and Photoluminescence Spectra of Polyurethanes in Solutions ········· **S24**
Thermal Properties of Polyurethanes ········· **S25**
Mechanical Properties of Polyurethanes ········· **S26**
NMR Spectra ········· **S27**
References ········· **S35**

Experimental Section

General and materials.

All reagents and solvents were purchased from FUJIFILM Wako Pure Chemical Corporation, Tokyo Kasei, Kanto Chemical, Merck, or Fluorochem. All reactions were carried out under nitrogen atmosphere unless otherwise noted. All reactions that require heating were conducted with an oil bath as the heat source. Flash silica gel column chromatography was conducted with Biotage Isolera or Biotage Select Flash systems using SHOKO-scientific Purif-Pack-EX cartridges. Recycling preparative gel permeation chromatography (GPC) was performed with a Japan Analytical Industry LaboACE. High-performance liquid chromatography (HPLC) was carried out using a SHIMADZU HPLC system equipped with InertSustain AQ-C18 columns (GL Sciences Inc.). Two types of columns were used: an analytical column (ID = 4.6 mm, L = 250 mm) and a preparative column (ID = 20 mm, L = 250 mm). Inhibitor-free anhydrous tetrahydrofuran was used as solvent during the synthesis of polymers. Telechelic poly(tetrahydrofuran)diol (M_n = 2,000) was dried in vacuo at 100 °C for 2 h before use. 4,4'-methylenebis(phenylisocyanate) and 1,4-butanediol were distilled under vacuum and stored over molecular sieves at 4 °C and at room temperature (r.t.), respectively.

^{1}H NMR spectra were measured with a JEOL JNM-ECZ400S spectrometer and all chemical shifts are reported on the δ-scale in ppm relative to the signal of tetramethylsilane (TMS at 0.00) or residual solvent protons (THF at 1.72) as the internal standard. Coupling constants (J) are quoted in Hz and relative intensities are reported. Proton-decoupled ^{13}C NMR spectra were acquired on a JEOL JNM-ECZ400S spectrometer and all chemical shifts are expressed in ppm using solvent as the internal standard ($CDCl_3$ at 77.16). Matrix Assisted Laser Desorption Ionization Time-of-Flight (MALDI-TOF) mass spectroscopy was performed with a SHIMADZU MALDI-8030. High-resolution electrospray ionization (ESI) mass spectra were measured with a Bruker Daltonics micrOTOF II. Size-exclusion chromatography (SEC) experiments were performed on a SHIMADZU Nexera GPC system equipped with a GPC KF–805L column (ID = 8.0 mm, L = 300 mm, particle size = 10 μm). Samples were injected using THF as the eluent at 40 °C and a flow rate of 1.0 mL/min. Data analysis was conducted on Labsolutions software (SHIMADZU) and molecular weights were calculated based on narrow-molecular-weight polystyrene calibration (1100–2,500,000 g/mol).

UV-VIS absorption spectra were recorded on a JASCO V-750 equipped with a temperature controller EHCS-932. Steady-state fluorescence spectra of solutions were recorded on a JASCO FP-8550 and the spectra were corrected for the detector nonlinearity. Time-resolved PL measurements were carried out with a Hamamatsu Photonics Quantaurus-Tau. Quantum efficiencies were measured with a Hamamatsu Photonics Quantaurus-QY. Steady-state PL spectra of polyurethane films during stretching experiments were monitored with an Ocean Optics OEPro-FL equipped with a Reflection/Backscattering Probe R400-7-UV-VIS; these spectra were not corrected. The excitation light at 365 nm was obtained using an Ocean Insight LLS-365 LED light source. The excitation light at 490 nm was obtained by passing the light of an Asahi Spectra CL-1501 equipped with an Asahi Spectra CL-H1-505-9-1 through a bandpass filter (Asahi Spectra HMX490). Stress-strain measurements were conducted under ambient conditions with a SHIMADZU AGS-100NX equipped with a 100 N load cell at strain rate of 300 mm/min. Thermogravimetric analyses (TGA) were performed under nitrogen with a SHIMADZU DTG-60 with a heating rate of 10 °C/min. Differential scanning calorimetry (DSC) measurements were conducted under N_2 on a Hitachi DSC7020 at heating and cooling rates of 10 ºC/min.

Femtosecond pump-probe system.

The transient absorption spectroscopy measurements were carried out using a home-made femtosecond pump−probe system. Yb laser (PHAROS PH2-10W, Light Conversion) was used to create fundamental light pulses at 1030 nm at a repetition rate of 1 kHz. The pulse energy was 200 μJ, and the pulse duration was approximately 290

fs. 65% of the fundamental beam was directed to ORPHEUS optical parametric amplifier (Light Conversion) to produce excitation pulses at the desired wavelength or utilized to generate SHG (515 nm) and THG (343 nm) for sample pumping. The rest of the beam was delivered to a delay stage then attenuated appropriately and focused onto liquid D_2O or a sapphire crystal to generate a stable white light continuum for sample probing. The probe light was introduced to polychromators equipped with a CMOS array (USP-PSMM-PK120, Unisoku) for the visible part of the spectrum and an InGaAs diode array (USP-NIR-PDA256, Unisoku) for the near-infrared (NIR) wavelengths. The measurements were carried out by comparing responses with and without excitation using a chopper synchronized with the fundamental laser pulses. The spectra were typically acquired by recording 2000 shots, that is, averaging over 2 s. Excitation energies were sufficiently lowered to avoid multiple exciton generation; this was verified by recording a series of measurements with different excitation energies for the same sample. No excitation energy dependence of the response was observed.

Global and target analysis.[S1] Global (singular value decomposition-based) and target (differential equation-based) analysis is accomplished using the Glotaran software package (http://glotaran.org).

Synthesis of Rotaxanes and Reference Compounds

The synthetic routes used to prepare **R-An**, **R-Te**, **Rot-Te**, **R-Pe**, and **Rot-Pe** are shown in Schemes S1, S2, S3, S4, and S5, respectively. 2-Iodo-1,4-dimethoxybenzene, 9,10-bis(trimethylsilylethynyl)anthracene, 2-(2-{2-[2-(4-iodophenoxy)ethoxy]ethoxy}ethoxy)ethanol, 5,12-bis(triisopropylsilylethynyl)tetracene, 1,5-bis{2-[2-(2-bromoethoxy)ethoxy]ethoxy}naphthalene, iodohydroquinone, compound **2**, compound **3**, and 6,13-diethynyl-6,13-dimethoxy-6,13-dihydropentacene were prepared according to reported procedures.[S2-S11]

Scheme S1

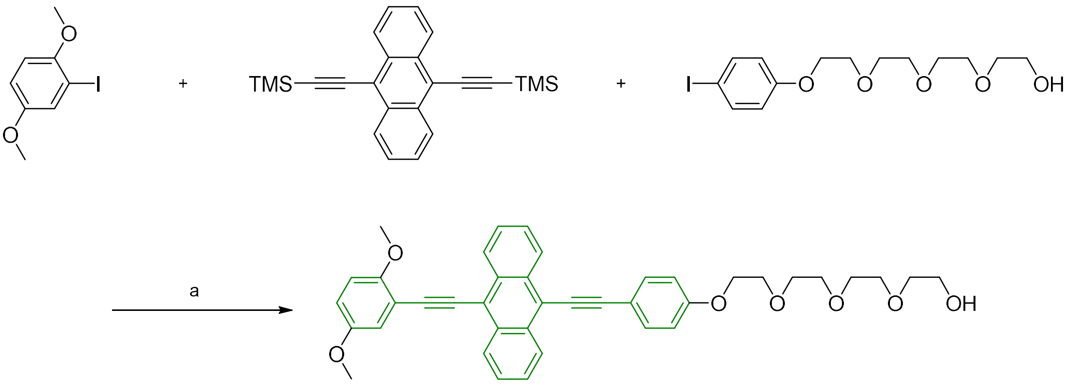

R-An

Conditions: (a) i) tetrabutylammonium fluoride, triethylamine, THF, r.t., 10 min, ii) $Pd(PPh_3)_4$, CuI, triethylamine, THF, 80 °C, 2 h.

R-An. To a mixture of 2-iodo-1,4-dimethoxybenzene (275 mg, 1.04 mmol), 9,10-bis(trimethylsilylethynyl)anthracene (386 mg, 1.04 mmol), 2-(2-{2-[2-(4-iodophenoxy)ethoxy]ethoxy}ethoxy)ethanol (413 mg, 1.04 mmol), triethylamine (20 mL), and THF (20 mL), a THF solution of tetrabutylammonium fluoride (ca. 1 mol/L, 2.60 mL, 2.60 mmol) was added and stirred under nitrogen atmosphere at r.t. for 10 min. Then, $Pd(PPh_3)_4$ (116 mg, 1.04×10^{-1} mmol) and CuI (40 mg, 0.21 mmol) were added and the reaction mixture was stirred under nitrogen atmosphere at 80 °C for 2 h. After cooling to r.t., the reaction mixture was poured into ethyl acetate (150 mL) and washed with 5% aq. HCl (150 mL), saturated aq. $NaHCO_3$ (150 mL), and saturated aq. NaCl (150 mL). The organic layer was separated, dried over $MgSO_4$, filtered, and the solvent was evaporated under reduced pressure. The crude product was purified by flash column chromatography on silica gel (eluent: gradient from dichloromethane to dichloromethane /acetone = 3:1 v/v) to afford **R-An** (245 mg, 0.373 mmol, 37%) as a yellow solid.

^{1}H NMR (400 MHz, $CDCl_3$): δ = 2.59 (t, J = 4.8 Hz, 1H), 3.62–3.64 (m, 2H), 3.70–3.79 (m, 10H), 3.87 (s, 3H), 3.92 (t, J = 4.4 Hz, 2H), 4.03 (s, 3H), 4.22 (t, J = 4.4 Hz, 2H), 6.95 (s, 2H), 7.01 (d, J = 7.6 Hz, 2H), 7.26 (s, 1H), 7.62–7.67 (m, 4H), 7.71 (d, J = 8.0 Hz, 2H), 8.67–8.70 (m, 2H), 8.81–8.83 (m, 2H).

^{13}C NMR (100 MHz, $CDCl_3$): δ = 56.00, 56.61, 61.83, 67.57, 69.72, 70.40, 70.65, 70.73, 70.90, 72.57, 85.58, 91.07, 98.98, 102.65, 112.07, 113.42, 114.93, 115.83, 115.97, 117.74, 118.52, 118.78, 126.73, 126.79, 127.29, 127.60, 132.01, 132.15, 133.23, 153.40, 154.94, 159.25.

HRMS (ESI): m/z: 653.2492 (calcd. $[M+Na]^+$ = 653.2510).

Scheme S2

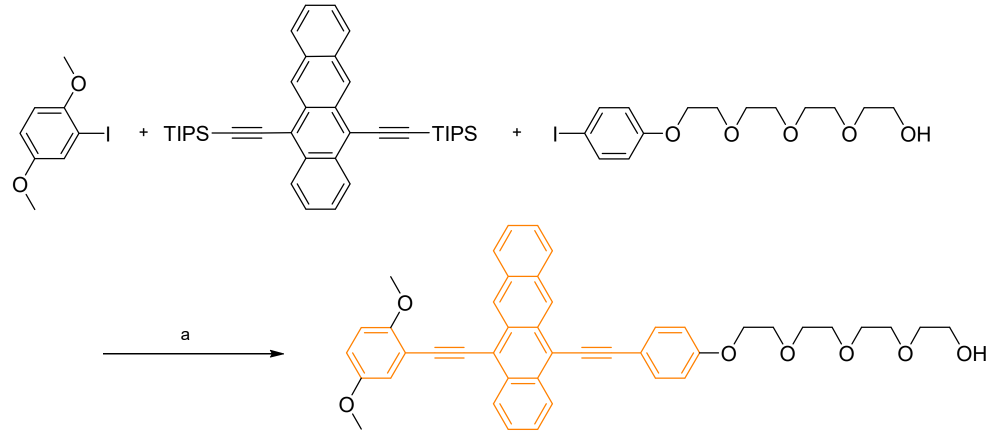

R-Te

Conditions: (a) i) tetrabutylammonium fluoride, triethylamine, THF, r.t., 10 min, ii) $Pd(PPh_3)_4$, CuI, triethylamine, THF, under N_2/H_2 atmosphere, 80 °C, 1.5 h.

R-Te. To a mixture of 2-iodo-1,4-dimethoxybenzene (132 mg, 5.00 × 10^{-1} mmol), 5,12-bis(trimethylsilylethynyl)tetracene (295 mg, 5.00 × 10^{-1} mmol), 2-(2-{2-[2-(4-iodophenoxy)ethoxy]ethoxy}ethoxy)ethanol (198 mg, 5.00 × 10^{-1} mmol), triethylamine (20 mL), and THF (20 mL), a THF solution of tetrabutylammonium fluoride (ca. 1 mol/L, 1.25 mL, 1.25 mmol) was added and stirred under nitrogen atmosphere at r.t. for 10 min. Then, $Pd(PPh_3)_4$ (116 mg, 0.104 mmol) and CuI (40 mg, 0.21 mmol) were added, and the reaction mixture was stirred under N_2/H_2 atmosphere at 80 °C for 1.5 h. After cooling to r.t., the reaction mixture was poured into chloroform (200 mL) and washed with 5% aq. HCl (150 mL), saturated aq. $NaHCO_3$ (150 mL), and saturated aq. NaCl (150 mL). The organic layer was separated, dried over $MgSO_4$, filtered, and the solvent was evaporated under reduced pressure. The crude product was purified by flash column chromatography on silica gel (eluent: gradient from dichloromethane to dichloromethane/acetone = 3:1 v/v), recycling GPC (eluent: chloroform), and HPLC (eluent: gradient from dichloromethane/MeOH = 3:17 v/v to dichloromethane/MeOH = 1:3 v/v) to afford **R-Te** (132 mg, 1.94 × 10^{-1} mmol, 39%) as a purple solid.

^{1}H NMR (400 MHz, $CDCl_3$): δ = 2.62 (t, J = 6.4 Hz, 1H), 3.62–3.64 (m, 2H), 3.68–3.79 (m, 10H), 3.89 (s, 3H), 3.90–3.93 (m, 2H), 4.09 (s, 3H), 4.21–4.24 (m, 2H), 6.96 (d, J = 2.0 Hz, 2H), 7.03 (d, J = 8.8 Hz, 2H), 7.31 (t, J = 1.6 Hz, 1H), 7.45–7.49 (m, 2H), 7.54–7.59 (m, 2H), 7.76 (d, J = 8.8 Hz, 2H), 8.08–8.12 (m, 2H), 8.63–8.67 (m, 1H), 8.76–8.79 (m, 1H), 9.26 (s, 1H), 9.44 (s, 1H).

^{13}C NMR (100 MHz, $CDCl_3$): δ = 56.02, 56.61, 61.86, 67.60, 69.75, 70.42, 70.68, 70.76, 70.93, 72.61, 86.29, 91.79, 99.94, 103.52, 112.00, 113.57, 114.97, 115.90, 116.01, 117.76, 118.39, 118.57, 125.87, 126.02, 126.50, 126.52, 126.57, 127.42, 127.70, 128.71, 129.92, 130.02, 132.12, 132.14, 132.19, 133.29, 153.42, 154.98, 159.27.

HRMS (ESI): m/z: 703.2664 (calcd. $[M+Na]^+$ = 703.2666).

Scheme S3

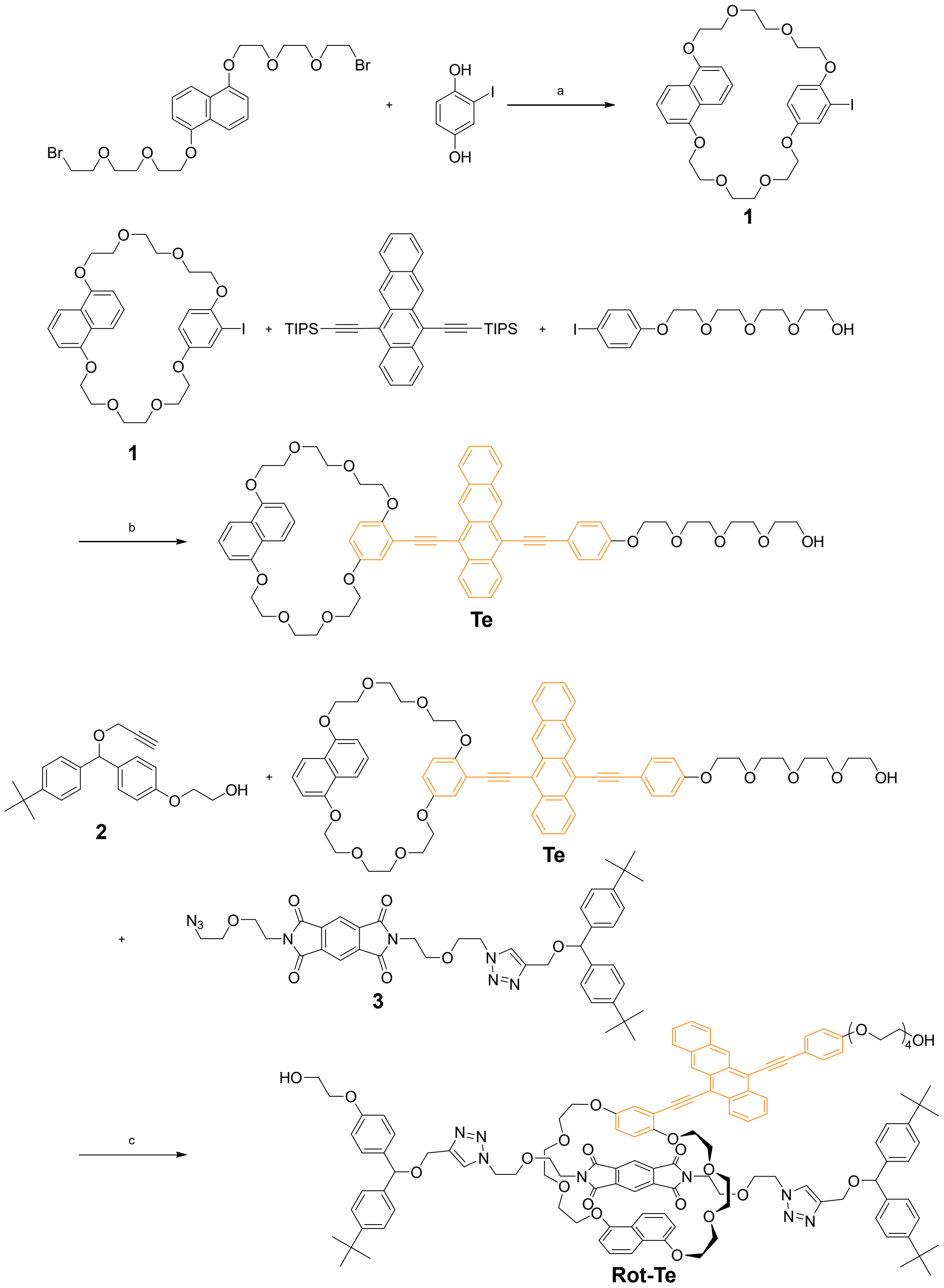

Conditions: (a) K_2CO_3, DMF, 80 °C, 23 h; (b) i) tetrabutylammonium fluoride, triethylamine, THF, r.t., 10 min, ii) $Pd(PPh_3)_4$, CuI, triethylamine, THF, under N_2/H_2 atmosphere, 80 °C, 2 h; (c) $CuSO_4$, sodium ascorbate, $CHCl_3$, H_2O, 5 °C, 5 h.

Compound 1. A solution of 1,5-bis{2-[2-(2-bromoethoxy)ethoxy]ethoxy}naphthalene (2.86 g, 5.20 mmol) and iodohydroquinone (1.23 g, 5.20 mmol) in DMF (100 mL) was added to a suspension of K_2CO_3 (14.37 g, 104.0 mmol) in DMF (250 mL) at 80 °C over the course of 6 h with vigorous stirring. After further stirring for 23 h at 80 °C, most of the DMF was evaporated under reduced pressure. The reaction mixture was then poured into ethyl acetate (150 mL) and washed with saturated aq. NH_4Cl (4 × 20 mL). The organic layer was separated, dried over $MgSO_4$, filtered and the solvent was evaporated under reduced pressure. The crude product was purified by flash column chromatography on silica gel (eluent: gradient from dichloromethane/acetone = 97:3 v/v to dichloromethane/acetone = 94:6 v/v) to afford compound **1** (0.56 g, 0.90 mmol, 17%) as a white solid.

^{1}H NMR (400 MHz, $CDCl_3$): δ = 3.54 (t, J = 4.8 Hz, 2H), 3.71–3.73 (m, 4H), 3.78–3.81 (m, 10H), 3.98–4.02 (m, 4H), 4.28–4.31 (m, 4H), 6.08 (dd, J = 8.8, 3.2 Hz, 1H), 6.25 (d, J = 8.8 Hz, 1H), 6.79–6.82 (m, 2H), 7.08 (d, J = 3.2 Hz, 1H), 7.23–7.28 (m, 2H), 7.84–7.88 (m, 2H).

^{13}C NMR (100 MHz, $CDCl_3$): δ = 67.68, 67.93, 68.36, 69.83, 69.86, 69.92, 70.27, 70.90, 71.13, 71.43, 71.48, 87.04, 105.65, 105.77, 114.56, 114.60, 114.69, 114.91, 125.26, 125.29, 126.06, 126.83, 152.21, 153.67, 154.36, 154.42.

MS (MALDI-TOF): m/z: 623.77 (calcd. [M] = 624.12).

Te. To a mixture of compound **1** (624 mg, 1.00 mmol), 5,12-bis(trimethylsilylethynyl)tetracene (396 mg, 1.00 mmol), 2-(2-{2-[2-(4-iodophenoxy)ethoxy]ethoxy}ethoxy)ethanol (589 mg, 1.00 mmol), triethylamine (20 mL), and THF (20 mL), a THF solution of tetrabutylammonium fluoride (ca. 1 mol/L, 2.50 mL, 2.50 mmol) was added and stirred under nitrogen atmosphere at r.t. for 10 min. Then, $Pd(PPh_3)_4$ (167 mg, 0.150 mmol) and CuI (29 mg, 0.15 mmol) were added and the reaction mixture was stirred under N_2/H_2 atmosphere at 80 °C for 2 h. After cooling to r.t., the reaction mixture was poured into chloroform (200 mL) and washed with 5% aq. HCl (150 mL), saturated aq. $NaHCO_3$ (150 mL), and saturated aq. NaCl (150 mL). The organic layer was separated, dried over $MgSO_4$, filtered, and the solvent was evaporated under reduced pressure. The crude product was purified by flash column chromatography on silica gel (eluent: gradient from dichloromethane to dichloromethane/acetone = 3:1 v/v), recycling GPC (eluent: chloroform), and HPLC (eluent: gradient from dichloromethane/MeOH = 1:4 v/v to dichloromethane/MeOH = 3:7 v/v) to afford **Te** (249 mg, 0.239 mmol, 24%) as a purple solid.

^{1}H NMR (400 MHz, $CDCl_3$): δ = 2.82 (br, 1H), 3.61–3.64 (m, 2H), 3.68–3.84 (m, 22H), 3.89–3.94 (m, 4H), 4.01–4.03 (m, 6H), 4.22 (t, J = 4.8 Hz, 4H), 4.31 (t, J = 4.4 Hz, 2H), 6.22 (dd, J = 8.8, 3.2 Hz, 1H), 6.33 (d, J = 8.8 Hz, 1H), 6.76–6.82 (m, 2H), 7.03 (d, J = 8.8 Hz, 2H), 7.07 (d, J = 3.2 Hz, 1H), 7.24–7.29 (m, 2H), 7.45–7.49 (m, 2H), 7.52–7.58 (m, 2H), 7.76 (d, J = 8.8 Hz, 2H), 7.89 (d, J = 8.4 Hz, 2H), 8.09–8.12 (m, 2H), 8.64–8.67 (m, 1H), 8.72–8.75 (m, 1H), 9.26 (s, 1H), 9.32 (s, 1H).

^{13}C NMR (100 MHz, $CDCl_3$): δ = 61.86, 67.61, 67.69, 67.78, 68.44, 69.11, 69.76, 69.88, 69.94, 70.05, 70.39, 70.68, 70.77, 70.95, 70.98, 71.18, 71.37, 71.49, 72.69, 86.27, 91.13, 100.38, 103.49, 105.62, 105.75, 113.75, 114.19, 114.58, 114.73, 115.01, 116.03, 116.30, 118.40, 118.64, 119.49, 125.33, 125.96, 125.99, 126.09, 126.46, 126.53, 126.61, 126.84, 127.44, 127.91, 128.72, 128.76, 129.92, 129.99, 132.18, 132.21, 132.24, 132.35, 133.33, 152.66, 154.00, 154.39, 154.46, 159.27.

HRMS (ESI): m/z: 1063.4236 (calcd. $[M+Na]^+$ = 1063.4239).

Rot-Te. A mixture of sodium ascorbate (112 mg, 0.565 mmol) and copper(II) sulfate (45.1 mg, 0.282 mmol) in water (1 mL) was added to a solution of compound **2** (127 mg, 0.377 mmol), compound **3** (293 mg, 0.377 mmol), and ring **Te** (129 mg, 0.124 mmol) in chloroform (1 mL) and the mixture was vigorously stirred at 5 °C for 5 h. The suspension was poured into a mixture of water (100 mL) and chloroform (150 mL) and washed with saturated aq. NaCl (100 mL). The organic layer was separated, dried over $MgSO_4$, filtered, and the solvent was evaporated under reduced pressure. The crude product was purified by flash column chromatography on silica gel (eluent: gradient from dichloromethane/acetone = 17:3 v/v to dichloromethane/acetone = 1:1 v/v), recycling GPC (eluent: chloroform), and

HPLC (eluent: gradient from dichloromethane/MeOH = 1:9 v/v to dichloromethane/MeOH = 1:4 v/v) to afford **Rot-Te** (40.7 mg, 1.93 × 10^{-2} mmol, 9%) as a purple solid.

^{1}H NMR (400 MHz, $CDCl_3$): δ = 1.24–1.25 (m, 27H), 2.21 (br, 1H), 2.66 (br, 1H), 3.21–4.49 (m, 60H), 4.57–4.62 (m, 4H), 5.41–5.45 (m, 2H), 6.03 (dd, J = 9.2, 2.0 Hz, 1H), 6.16 (dd, J = 8.8, 2.8 Hz, 1H), 6.24 (t, J = 7.2 Hz, 1H), 6.31–6.34 (m, 1H), 6.51–6.52 (m, 1H), 6.69–6.75 (m, 1H), 6.79–6.82 (m, 3H), 7.04 (d, J = 8.8 Hz, 2H), 7.20–7.29 (m, 16H), 7.47–7.58 (m, 5H), 7.67 (d, J = 14.0 Hz, 1H), 7.78 (d, J = 8.8 Hz, 2H), 7.98 (br, 2H), 8.09–8.12 (m, 1H), 8.28–8.30 (m, 1H), 8.61–8.63 (m, 1H), 8.66–8.68 (m, 1H), 9.19 (s, 1H), 9.28 (s, 1H).

^{13}C NMR (100 MHz, $CDCl_3$): δ = 31.46, 34.57, 36.48, 36.64, 36.74, 50.17, 50.34, 61.50, 61.91, 62.34, 62.37, 62.46, 62.50, 67.71, 67.83, 67.90, 68.22, 68.53, 68.55, 69.25, 69.52, 69.82, 69.91, 70.00, 70.49, 70.76, 70.83, 71.02, 71.15, 72.67, 82.42, 82.44, 82.48, 82.84, 82.89, 86.09, 91.73, 99.33, 104.08, 105.34, 105.54, 111.20, 112.56, 114.14, 114.30, 114.50, 115.14, 115.80, 117.21, 117.30, 117.96, 118.30, 118.89, 123.64, 123.76, 124.18, 124.24, 125.40, 125.68, 125.79, 126.09, 126.33, 126.84, 126.91, 127.41, 128.06, 128.59, 129.22, 129.73, 129.88, 132.21, 132.32, 132.48, 132.51, 133.44, 134.35, 134.46, 134.64, 134.66, 138.94, 139.00, 145.52, 145.59, 145.66, 145.73, 150.34, 150.37, 151.99, 153.09, 153.11, 154.00, 158.11, 159.51, 166.72, 166.86.

HRMS (ESI): m/z: 1100.4822 (calcd. $[M+2Na]^{2+}$ = 1100.4830).

Scheme S4

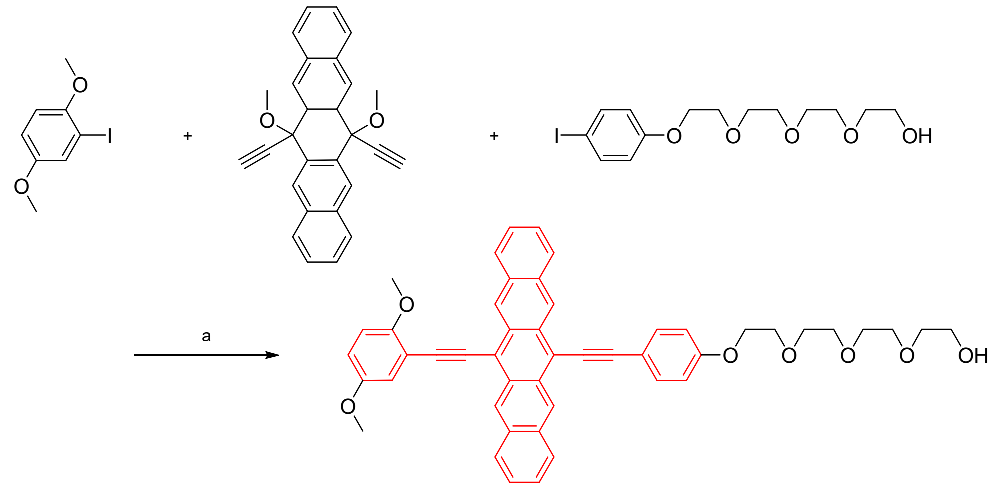

Conditions: (a) i) $Pd(PPh_3)_4$, CuI, triethylamine, THF, 80ºC, 2 h, ii) $SnCl_2$, triethylamine, THF, r.t., 30 min.

R-Pe. To a mixture of 2-iodo-1,4-dimethoxybenzene (79 mg, 3.0 × 10^{-1} mmol), 6,13-diethynyl-6,13-dimethoxy-6,13-dihydropentacene (117 mg, 3.00 × 10^{-1} mmol), 2-(2-{2-[2-(4-iodophenoxy)ethoxy]ethoxy}ethoxy)ethanol (119 mg, 3.00 × 10^{-1} mmol), triethylamine (20 mL), and THF (20 mL), $Pd(PPh_3)_4$ (16 mg, 1.5 × 10^{-2} mmol) and CuI (5 mg, 3.0 × 10^{-2} mmol) were added and the reaction mixture was stirred under the nitrogen atmosphere at 80 °C for 2 h. After cooling to r.t., large excess amounts of $SnCl_2$ (1.71 g, 9.00 mmol) were added and the reaction mixture was stirred at r.t. for 30 min. The reaction mixture was poured into chloroform (200 mL) and washed with 5% aq. HCl (150 mL), saturated aq. $NaHCO_3$ (150 mL), and saturated aq. NaCl (150 mL). The organic layer was separated, dried over $MgSO_4$, filtered through celite, and the solvent was evaporated under reduced pressure. The crude product was purified by flash column chromatography on silica gel (eluent: gradient from dichloromethane to dichloromethane/acetone = 3:1 v/v), recycling GPC (eluent: chloroform), and HPLC (eluent: gradient from dichloromethane/MeOH = 1:9 v/v to dichloromethane/MeOH = 3:17 v/v) to afford **R-Pe** (29 mg, 4.0 × 10^{-2} mmol,

13%) as a blue solid.

^{1}H NMR (400 MHz, $CDCl_3$): δ = 2.76 (br, 1H), 3.63–3.65 (m, 2H), 3.71–3.81 (m, 10H), 3.92 (s, 3H), 3.94 (t, J = 4.8 Hz, 2H), 4.13 (s, 3H), 4.26 (t, J = 4.8 Hz, 2H), 6.99 (s, 2H), 7.07 (d, J = 8.8 Hz, 2H), 7.36 (s, 1H), 7.38–7.42 (m, 4H), 7.82 (d, J = 8.8 Hz, 2H), 7.99–8.02 (m, 4H), 9.18 (s, 2H), 9.35 (s, 2H).

^{13}C NMR (100 MHz, $CDCl_3$): δ = 56.12, 56.67, 61.92, 67.66, 69.83, 70.48, 70.75, 70.83, 71.01, 72.63, 87.19, 92.73, 101.19, 104.74, 111.99, 113.71, 115.06, 115.91, 116.19, 117.85, 118.11, 118.28, 125.92, 126.10, 126.56, 128.82, 128.86, 130.19, 130.24, 132.21, 132.27, 133.39, 153.48, 155.05, 159.35.

HRMS (ESI): m/z: 753.2812 (calcd. $[M+Na]^+$ = 753.2823).

Scheme S5

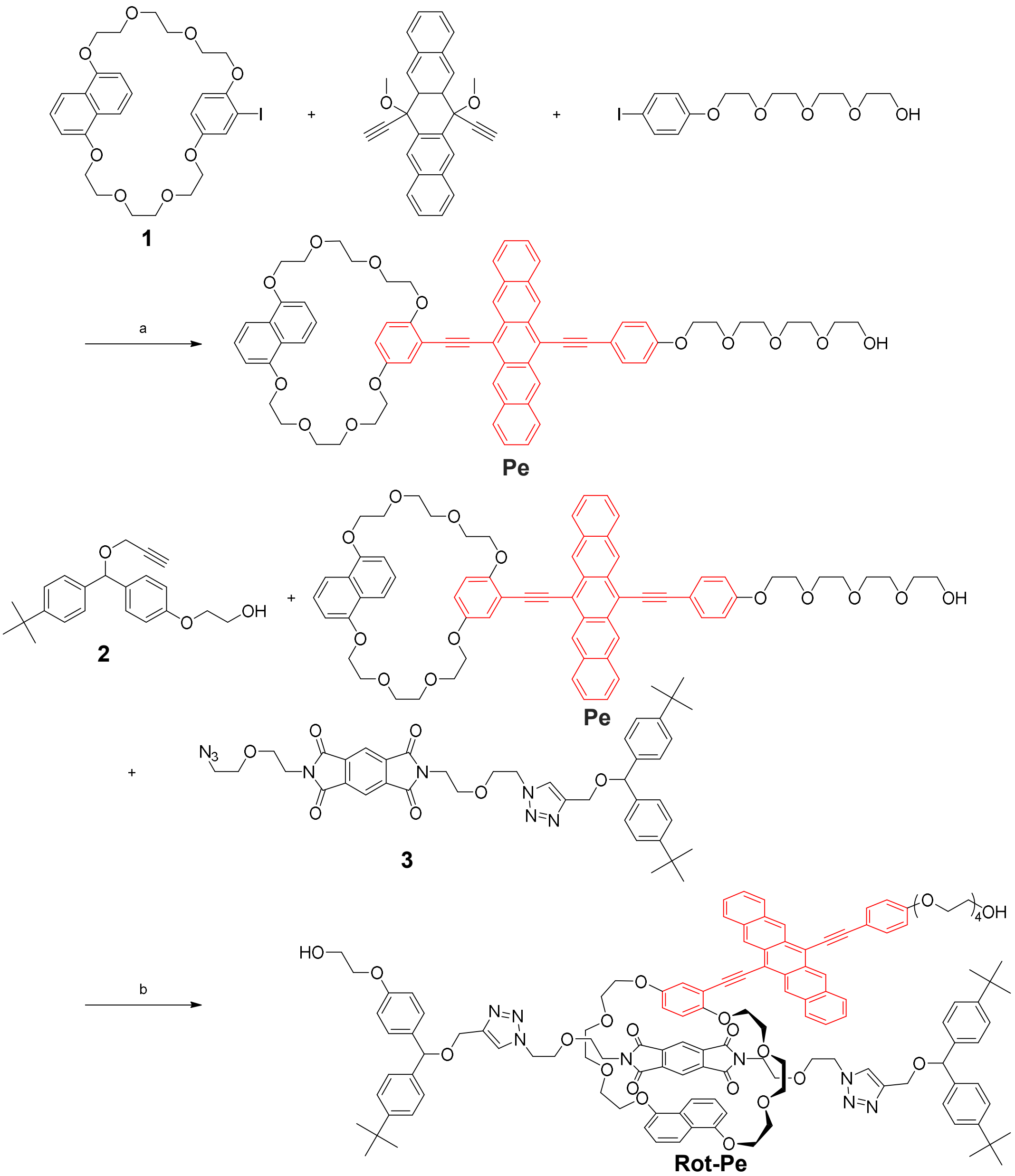

Conditions: (a) i) $Pd(PPh_3)_4$, CuI, THF, Et_3N, 60ºC, 3 h, ii) $SnCl_2$, THF, Et_3N, r.t., 10 min; (b) $CuSO_4$, sodium ascorbate, $CHCl_3$, H_2O, 5ºC, 4 h.

Pe. To a mixture of compound **1** (500 mg, 1.28 mmol), 6,13-diethynyl-6,13-dimethoxy-6,13-dihydropentacene (800 mg, 1.28 mmol), 2-(2-{2-[2-(4-iodophenoxy)ethoxy]ethoxy}ethoxy)ethanol (507 mg, 1.28 mmol), triethylamine (20 mL), and THF (20 mL), $Pd(PPh_3)_4$ (120 mg, 1.08 × 10^{-1} mmol) and CuI (40 mg, 2.2 × 10^{-1} mmol) were added and the reaction mixture was stirred under the nitrogen atmosphere at 60 °C for 3 h. After cooling to r.t., large excess amounts of $SnCl_2$ (4.86 g, 25.6 mmol) were added and the reaction mixture was stirred at r.t. for 10 min. The reaction mixture was poured into chloroform (200 mL) and washed with 5% aq. HCl (150 mL), saturated aq. $NaHCO_3$ (150 mL), and saturated aq. NaCl (100 mL). The organic layer was separated, dried over $MgSO_4$, filtered through celite, and the solvent was evaporated under reduced pressure. The crude product was purified by flash column chromatography on silica gel (eluent: gradient from dichloromethane to dichloromethane/acetone = 3:1 v/v), recycling GPC (eluent: chloroform), and HPLC (eluent: gradient from dichloromethane/MeOH = 1:9 v/v to dichloromethane/MeOH = 3:17 v/v) to afford **Pe** (192 mg, 1.79 × 10^{-1} mmol, 14%) as a blue solid.

^{1}H NMR (400 MHz, $CDCl_3$): δ = 2.74 (br, 1H), 3.62–3.65 (m, 2H), 3.71–3.85 (m, 22H), 3.91–3.94 (m, 4H), 3.99–4.04 (m, 4H), 4.12 (t, J = 5.2 Hz, 2H), 4.20–4.26 (m, 4H), 4.34 (t, J = 4.4 Hz, 2H), 6.23 (dd, J = 8.8, 2.8 Hz, 1H), 6.43 (d, J = 8.8 Hz, 1H), 6.78–6.84 (m, 2H), 7.07 (d, J = 8.8 Hz, 2H), 7.14 (d, J = 2.8 Hz, 1H), 7.25–7.32 (m, 2H), 7.38–7.43 (m, 4H), 7.82 (d, J = 8.8 Hz, 2H), 7.89–7.92 (m, 2H), 8.02–8.05 (m, 4H), 9.23 (s, 2H), 9.30 (s, 2H).

^{13}C NMR (100 MHz, $CDCl_3$): δ = 61.91, 67.65, 67.68, 67.81, 68.33, 69.43, 69.81, 69.88, 69.89, 69.98, 70.09, 70.47, 70.74, 70.81, 70.97, 71.00, 71.20, 71.31, 71.55, 72.62, 87.16, 91.90, 101.66, 104.72, 105.62, 105.78, 114.09, 114.60, 114.64, 114.74, 115.06, 116.09, 116.14, 118.11, 118.34, 119.59, 125.33, 125.37, 125.94, 125.98, 126.11, 126.51, 126.86, 128.80, 128.88, 130.23, 130.24, 132.24, 132.28, 133.38, 152.75, 153.94, 154.40, 154.49, 159.35.

HRMS (ESI): m/z: 1113.4410 (calcd. $[M+Na]^+$ = 1113.4396).

Rot-Pe. A mixture of sodium ascorbate (25 mg, 1.3 × 10^{-1} mmol) and copper(II) sulfate (7 mg, 4 × 10^{-2} mmol) in water (1 mL) was added to a solution of compound **2** (97 mg, 0.286 mmol), compound **3** (222 mg, 0.286 mmol), and ring **Pe** (153 mg, 0.143 mmol) in chloroform (0.5 mL) and the mixture was vigorously stirred at 5 °C for 4 h. The suspension was poured into a mixture of water (100 mL) and chloroform (150 mL) and washed with saturated aq. NaCl (100 mL). The organic layer was separated, dried over $MgSO_4$, filtered, and the solvent was evaporated under reduced pressure. The crude product was purified by flash column chromatography on silica gel (eluent: gradient from dichloromethane/acetone = 17:3 v/v to dichloromethane/acetone = 1:1 v/v), recycling GPC (eluent: chloroform), and HPLC (eluent: gradient from dichloromethane/MeOH = 1:9 v/v to dichloromethane/MeOH = 3:17 v/v) to afford **Rot-Pe** (24 mg, 1.1 × 10^{-2} mmol, 7.6%) as a blue solid.

^{1}H NMR (400 MHz, $CDCl_3$): δ = 1.23–1.25 (m, 27H), 2.12–2.22 (m, 1H), 2.68–2.72 (m, 1H), 3.08–4.61 (m, 64H), 5.38–5.45 (m, 2H), 6.06 (dd, J = 9.2, 2.8 Hz, 1H), 6.15 (dd, J = 9.2, 2.8 Hz, 1H), 6.24 (t, J = 7.6 Hz, 1H), 6.34 (t, J = 6.8 Hz, 1H), 6.59–6.60 (m, 1H), 6.69–6.84 (m, 4H), 7.07–7.10 (m, 2H), 7.18–7.29 (m, 16H), 7.42–7.49 (m, 5H), 7.66–7.70 (m, 1H), 7.86 (d, J = 8.8 Hz, 2H), 8.02–8.07 (m, 4H), 8.24–8.25 (m, 2H), 9.21 (s, 2H), 9.28 (s, 2H).

^{13}C NMR (100 MHz, $CDCl_3$): δ = 31.44, 34.56, 36.43, 36.63, 36.66, 50.05, 50.33, 61.47, 61.90, 62.26, 62.34, 62.38, 62.46, 67.70, 67.76, 68.02, 68.16, 68.51, 68.54, 69.21, 69.50, 69.80, 69.95, 70.04, 70.45, 70.74, 70.82, 71.00, 71.19, 72.70, 82.33, 82.42, 82.78, 82.85, 86.96, 92.36, 100.54, 105.31, 105.37, 105.62, 111.61, 112.73, 114.13, 114.27, 114.44, 114.47, 115.17, 115.92, 117.06, 117.14, 117.65, 118.29, 118.39, 118.56, 118.58, 123.59, 123.78, 124.24, 125.38, 125.40, 125.66, 125.74, 126.06, 126.30, 126.81, 126.89, 128.56, 128.70, 129.29, 130.15, 132.39, 132.54, 133.49, 134.32, 134.44, 134.62, 138.88, 138.91, 138.96, 145.41, 145.49, 145.60, 145.68, 150.31, 150.33, 150.37, 151.98, 153.02, 153.05, 154.01, 158.05, 158.09, 159.51, 166.72, 166.93.

HRMS (ESI): m/z: 1125.9896 (calcd. $[M+2Na]^{2+}$ = 1125.4908).

Synthesis of Polyurethanes

Rot-Te-PU and **Rot-Pe-PU** were obtained according to Schemes S6, and S7, respectively.

Scheme S6

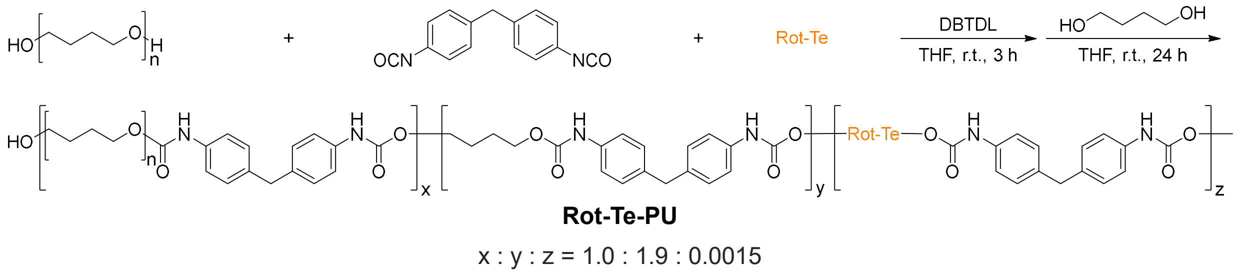

Rot-Te-PU. Dibutyltin dilaurate (DBTDL, 4 drops) was added to a stirred mixture of **Rot-Te** (10 mg, 4.9 μmol), telechelic poly(tetrahydrofuran)diol (M_n = 2,000, 3.00 g, 1.50 mmol), and 4,4′-methylenebis-(phenylisocyanate) (1.27 g, 5.08 mmol) in THF (30 mL) and the mixture was stirred at r.t. for 3 h. A solution of 1,4-butanediol (297 mg, 3.30 mmol) in THF (10 mL) was then added and the reaction mixture was stirred at r.t. for additional 24 h. MeOH (5 mL) was added to the reaction mixture and the reaction mixture was poured into MeOH (1500 mL) after stirring for another 30 min. The pink precipitate was collected by filtration and was redissolved in THF (100 mL). The THF solution was filtrated through a cotton filter and poured into hexane (1600 mL). The precipitate was filtered off and dried in vacuo for 15 h at r.t. to afford **Rot-Te-PU** as a pink rubbery solid (4.24 g, 94%, M_n = 132,000). The x:y and x:z ratios were determined by ^{1}H NMR spectroscopy and the fraction of **Rot-Te** in the monomer feed.

Scheme S7

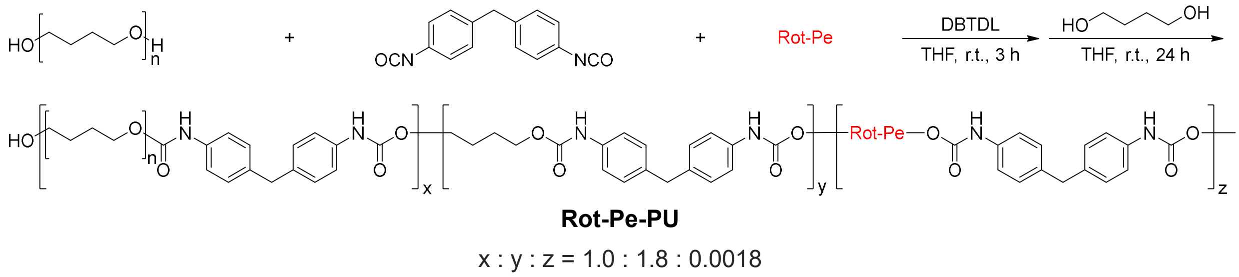

Rot-Pe-PU. DBTDL (4 drops) was added to a stirred mixture of **Rot-Pe** (3.0 mg, 1.4 μmol), telechelic poly(tetrahydrofuran)diol (M_n = 2,000, 1.50 g, 0.75 mmol), and 4,4′-methylenebis-(phenylisocyanate) (0.63 g, 2.52 mmol) in THF (30 mL) and the mixture was stirred at r.t. for 3 h. A solution of 1,4-butanediol (149 mg, 1.65 mmol) in THF (10 mL) was then added and the reaction mixture was stirred at r.t. for additional 24 h. MeOH (5 mL) was added to the reaction mixture and the reaction mixture was poured into MeOH (1500 mL) after stirring for another 30 min. The pale blue precipitate was collected by filtration and was redissolved in THF (100 mL). The THF solution was filtrated through a cotton filter and poured into hexane (1600 mL). The precipitate was filtered off and dried in vacuo for 24 h at r.t. to afford **Rot-Pe-PU** as a pale blue rubbery solid (2.11 g, 47%, M_n = 61,000). The x:y and x:z ratios were determined by ^{1}H NMR spectroscopy and the fraction of **Rot-Pe** in the monomer feed.

Preparation of Polyurethane Films

Preparation of Rot-Te-PU and Rot-Pe-PU films. 300 mg of the polyurethane (**Rot-Te-PU** or **Rot-Pe-PU**) was dissolved in THF (8 mL) and each solution was divided between two square poly(tetrafluoroethylene) molds (35 × 35 × 4.0 mm). The molds were placed under an inverted funnel so that the evaporation rate was controlled. The solvent was evaporated for more than 12 h under ambient conditions and the resulting films were further dried in vacuo at r.t. for 3 h. The films thus obtained were smooth and transparent or opaque. The thicknesses of the films were 60–80 μm, which were measured by a digital caliper.

Qualitative Jablonski Diagrams

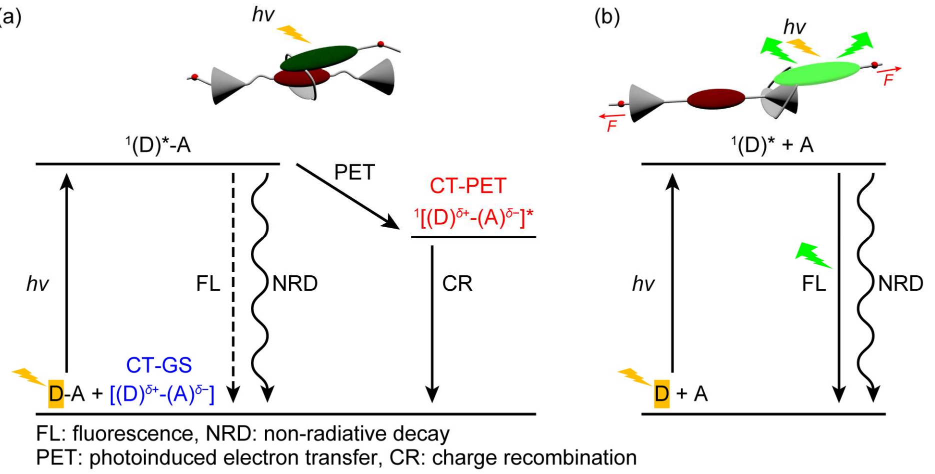

Fig. S1 Jablonski diagrams of the rotaxane mechanophores (a) before and (b) after mechanical activation.

Method for Estimating the Energy of the CT State[S12]

The free energy change (ΔG) for the charge separation (ΔG_{CS}) and charge recombination (ΔG_{CR}) were calculated employing the following relations:

$$-\Delta G_{\mathrm{CR}} = E_{\mathrm{ox}} - E_{\mathrm{red}} + \Delta G\mathrm{s}$$
$$-\Delta G_{\mathrm{CS}} = \Delta E_{0\text{-}0} - (-\Delta G_{\mathrm{CR}})$$
$$\Delta G\mathrm{s} = e^2/(4\pi\epsilon_0)[(1/(2R^+) + 1/(2R^-) - 1/R_{\mathrm{cc}})(1/\epsilon_{\mathrm{s}}) - (1/(2R^+) + 1/(2R^-))(1/\epsilon_{\mathrm{r}})]$$

, where E_{ox} and E_{red} are the first oxidation potential of fluorophore and the first reduction potential of PMDI, e is the charge of the electron, ϵ_{s} and ϵ_{r} are static dielectric constants of solvent used and when measured the redox potentials, respectively, R^+ and R^- are the values of hard-sphere radii of fluorophore and PMDI, R_{cc} is center-to-center distance between fluorophore and PMDI, and ϵ_0 is the permittivity of free space.

In this study, the values of R^+ and R^- are the radii of the molecules corresponding to the long-axis direction in the corresponding molecule and ϵ_{r} is the dielectric constant of the solvent used for cyclic voltammetry of the acceptor. The values used in the calculations and calculated $-\Delta G_{\mathrm{CR}}$ values are given below.

	E_{ox} (vs Fc$^+$/Fc)	E_{red} (vs Fc$^+$/Fc)	$\Delta E_{0\text{-}0}$ (eV)	R^+ (Å)	R^- (Å)	R_{cc} (Å)	ϵ_{s}	ϵ_{r}	$-\Delta G_{\mathrm{CR}}$ (eV in toluene)
**Rot-An**	0.68[S13]	−1.37[S14]	2.7	8.3	3.4	9.9	2.38[a]	8.93[b]	2.4
**Rot-Te**	0.47[S15]	−1.37[S14]	2.2	8.3	3.4	9.9	2.38[a]	8.93[b]	2.1
**Rot-Pe**	0.26[S16]	−1.37[S14]	1.9	8.3	3.4	9.9	2.38[a]	8.93[b]	1.9

[a]In toluene.[S17] [b]In dichloromethane.[S17]

^{1}H NMR Spectra of Rotaxanes

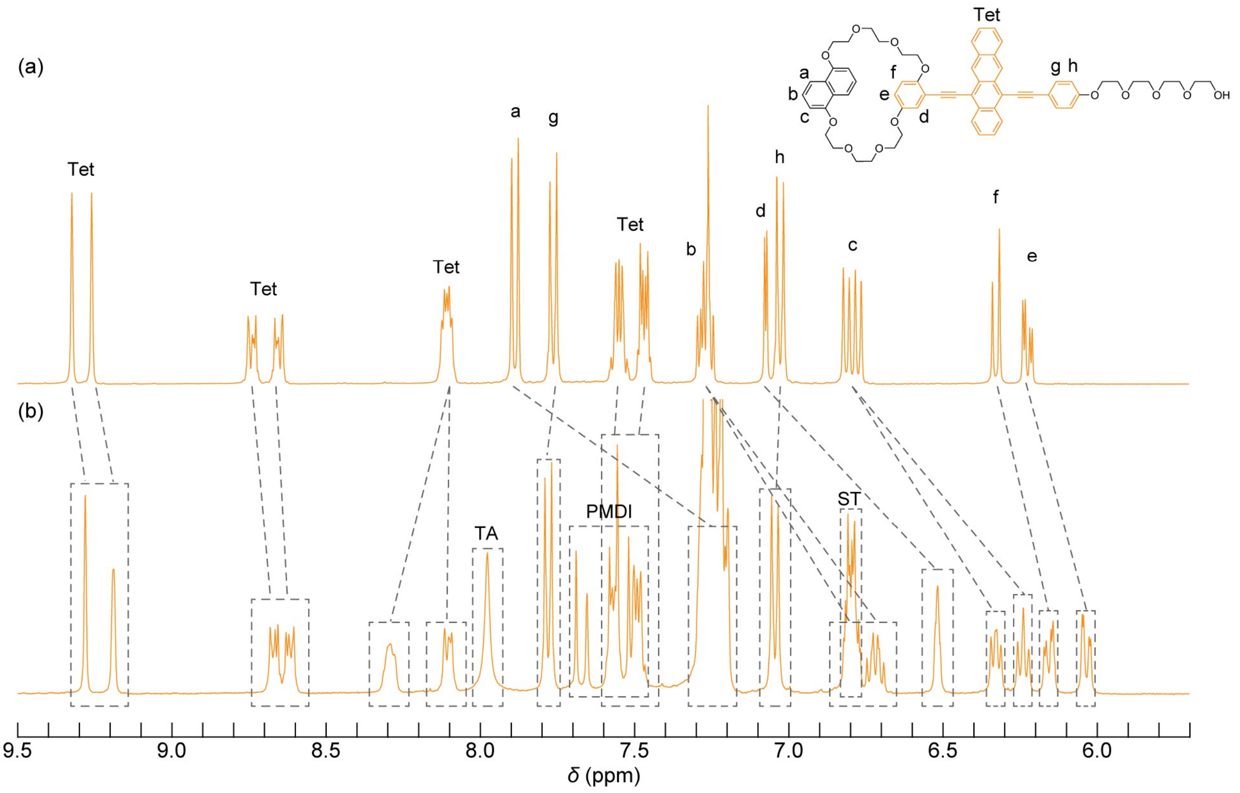

Fig. S2 Partial ^{1}H NMR spectra of (a) ring compound **Te** and (b) rotaxane **Rot-Te**. The abbreviations "ST", "TA", "PMDI" indicate signals corresponding to the stoppers, triazole, and quencher moieties, respectively.

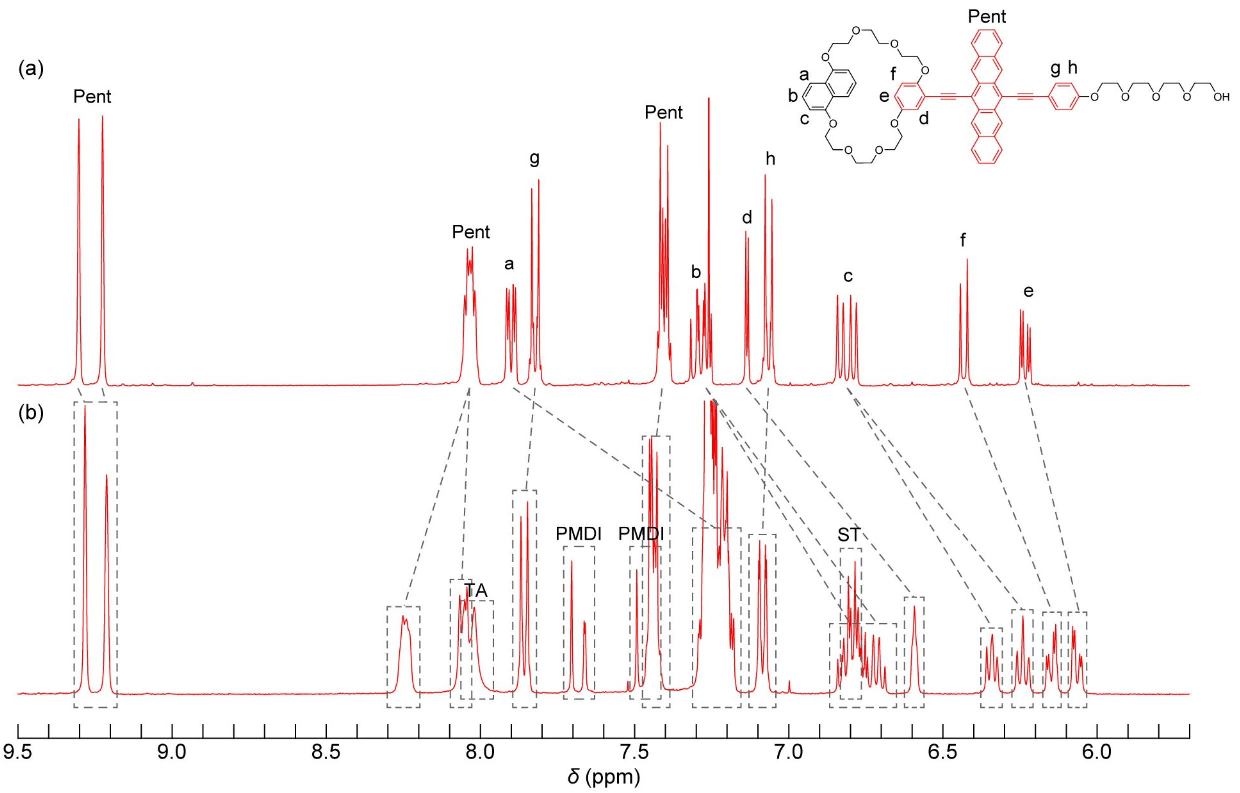

Fig. S3 Partial ^{1}H NMR spectra of (a) ring compound **Pe** and (b) rotaxane **Rot-Pe**. The abbreviations “ST”, “TA”, “PMDI” indicate signals corresponding to the stoppers, triazole, and quencher moieties, respectively.

Quantum Yields and Fluorescence Lifetimes of References and Rotaxanes in Toluene

Table S1. Fluorescence quantum yields (Φ) of rotaxanes and reference compounds in toluene along with excitation wavelength (λ_{ex}).

	Φ (%)	λ_{ex} (nm)
**R-An**	91	430
**Rot-An**	< 1	430
**R-Te**	73	525
**Rot-Te**	1	525
**R-Pe**	37	615
**Rot-Pe**	14	615

Table S2. Fluorescence lifetimes (τ) of reference compounds in toluene solution ($c = 1 \times 10^{-5}$ M) along with excitation (λ_{ex}) and monitor (λ_{emi}) wavelengths.

	τ (ns)	λ_{ex} (nm)	λ_{emi} (nm)
**R-An**	2.63	405	495
**R-Te**	5.90	365	580
**R-Pe**	7.89	630	690

Titration Experiments for Estimating the Percentage of CT Complex Formation for Rot-An

1. Materials

In the absorption spectrum of **Rot-An**, a tail band was observed in the long-wavelength region, which is attributed to charge transfer (CT) interactions between the fluorophore donor and quencher acceptor. However, not all rotaxanes undergo CT complex formation. To estimate the proportion of rotaxanes that form CT complexes, the percentage of CT complex-forming rotaxanes (P_{CT}) was defined as follows:

$$P_{CT}\ (\%) = 100 \times (\varepsilon_{Rot\text{-}An} - \varepsilon_{D})/(\varepsilon_{D\text{–}A} - \varepsilon_{D}) \qquad (1)$$

, where $\varepsilon_{Rot\text{-}An}$ denotes the molar absorption coefficient of the rotaxane at the CT absorption band, $\varepsilon_{D\text{–}A}$ represents that of the fully formed donor–acceptor CT complex, and ε_{D} is the molar absorption coefficient of the uncomplexed donor moiety at the same wavelength. To determine $\varepsilon_{D\text{–}A}$, titration experiments were conducted using the cyclic donor **An** and acceptor PMDI derivative **Ax** (as shown in Fig. S3).

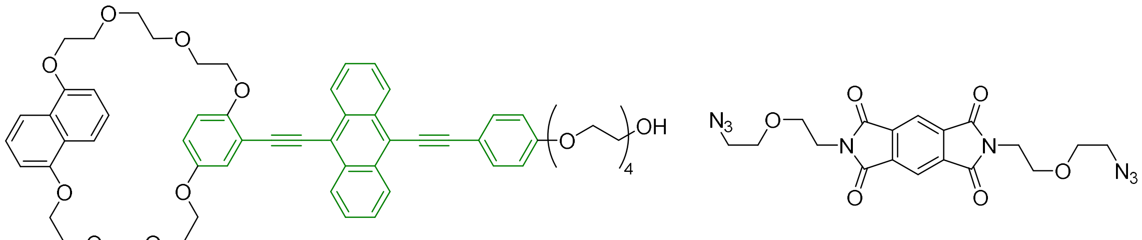

Fig. S4 Molecular structures of donor **An** and acceptor **Ax** for titration experiments.

2. Calculation processes of molar absorption coefficient of CT complexes ($\varepsilon_{D\text{–}A}$)

In a mixed solution of **An** and **Ax**, the absorbance (A) is described by the following equation, which incorporates the molar absorption coefficient of the CT complex, $\varepsilon_{D\text{–}A}$:

$$A = (\varepsilon_{D\text{–}A}[D\text{–}A] + \varepsilon_{D}[D] + \varepsilon_{A}[A])l \qquad (2)$$

, where [D–A], [D], and [A] represent the concentration of the CT complex, the free donor, and the free acceptor in solution, respectively, and ε_{A} is the molar absorption coefficient of the acceptor **Ax**. Given that **Ax** does not absorb at 570 nm and that a 1 cm pathlength optical cell (l = 1 cm) is used, the equation can be simplified as follows:

$$A\ (\text{at 570 nm}) = \varepsilon_{D\text{–}A}[D\text{–}A] + \varepsilon_{D}[D] \qquad (3)$$

The association constant (K) for CT complex formation is defined by the following equilibrium equation:

$$K = [D\text{–}A]/[D][A] \quad (4)$$

Because [D–A] is dependent on the initial concentrations of **An** ($[D]_0$) and **Ax** ($[A]_0$), the concentration of the CT complex can be expressed as a function of $[D]_0$ and $[A]_0$:

$$[D\text{–}A] = \frac{([D]_0+[A]_0+1/K)-\sqrt{([D]_0+[A]_0+1/K)^2-4[D]_0[A]_0}}{2} \qquad (5)$$

Substituting equation (5) into equation (3), the absorbance at 570 nm can be expressed as:

$$A\ (\text{at 570 nm}) = \epsilon_{D}[D]_0 + (\epsilon_{D-A} - \epsilon_{D})\frac{([D]_0+[A]_0+1/K)-\sqrt{([D]_0+[A]_0+1/K)^2-4[D]_0[A]_0}}{2} \qquad (6)$$

In equation (6), $\varepsilon_{D\text{-}A}$ and K were treated as fitting parameters. These values were determined by fitting the absorbance data obtained at various initial concentrations of **Ax** ($[A]_0$) in the titration experiments.

3. The results of the titration experiments

To determine ε_{D-A}, a 6.64 mM solution of the donor **An** in $CHCl_3$ was used as the base solution. A single, concentrated titrant solution was also prepared by dissolving the acceptor **Ax** in the same 6.64 mM **An** solution, resulting in an **Ax** concentration of 166 mM. Titrations were performed by incrementally adding aliquots of the **Ax**/**An** mixed solutions to the base **An** solution. This procedure ensured that the concentration of **An** remained constant throughout the titration, while only the concentration of **Ax** was increased. After each addition, absorption spectra were recorded at 25 °C (Fig. S5 (a)). The experimental absorbance data were then fitted to Equation (6) using the least-squares method to obtain the two fitting parameters: the association constant K and the molar absorption coefficient ε_{D-A} (Fig. S5 (b)). The molar absorption coefficient of the rotaxane, $\varepsilon_{Rot\text{-}An}$, was determined from a 1 mM $CHCl_3$ solution of **Rot-An** ($\varepsilon_{Rot\text{-}An}$ = 194 M^{-1} cm^{-1}). The values of K, ε_{D-A}, and P_{CT} were determined to be 2.20 M^{-1}, 1028 M^{-1} cm^{-1}, and 18%, respectively.

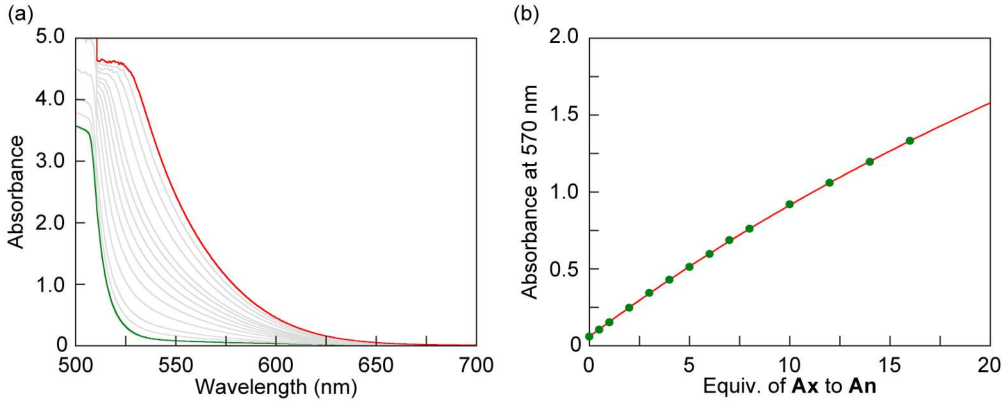

Fig. S5 (a) Absorption spectra obtained during the titration of a 6.64 × 10^{-3} M solution of **An** with **Ax** at 25 °C in $CHCl_3$. (b) Titration curve showing the change in absorbance with increasing concentrations of **Ax**, plotted using data from Table S3.

Table S3. Raw data corresponding to absorbance changes induced by titration of **An** with **Ax** in $CHCl_3$ at 25 °C, obtained from the spectra in Fig. S5.

Donor concentration $[D]_0$ (M)	Acceptor concentration $[A]_0$ (M)	Absorbance at 570 nm A
0.00664	0.00000	0.059822
0.00664	0.00332	0.103992
0.00664	0.00664	0.152320
0.00664	0.01328	0.246780
0.00664	0.01992	0.342832
0.00664	0.02656	0.428393
0.00664	0.03320	0.512224
0.00664	0.03984	0.597102
0.00664	0.04648	0.686343
0.00664	0.05312	0.761247
0.00664	0.06640	0.920114
0.00664	0.07968	1.059720
0.00664	0.09296	1.195940
0.00664	0.10624	1.332520

Comparison of the Absorption Spectra of Rot-An in Chloroform and Toluene

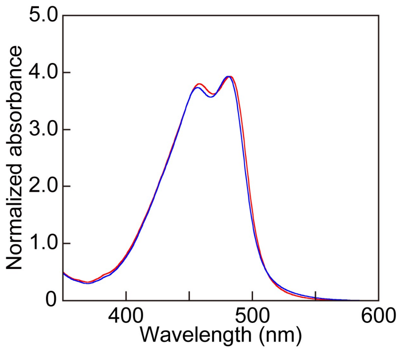

Fig. S6 Absorption spectra of **Rot-An** in chloroform (blue) and toluene (red) with concentrations of 1.0×10^{-5} M.

Transient Absorption Spectroscopy

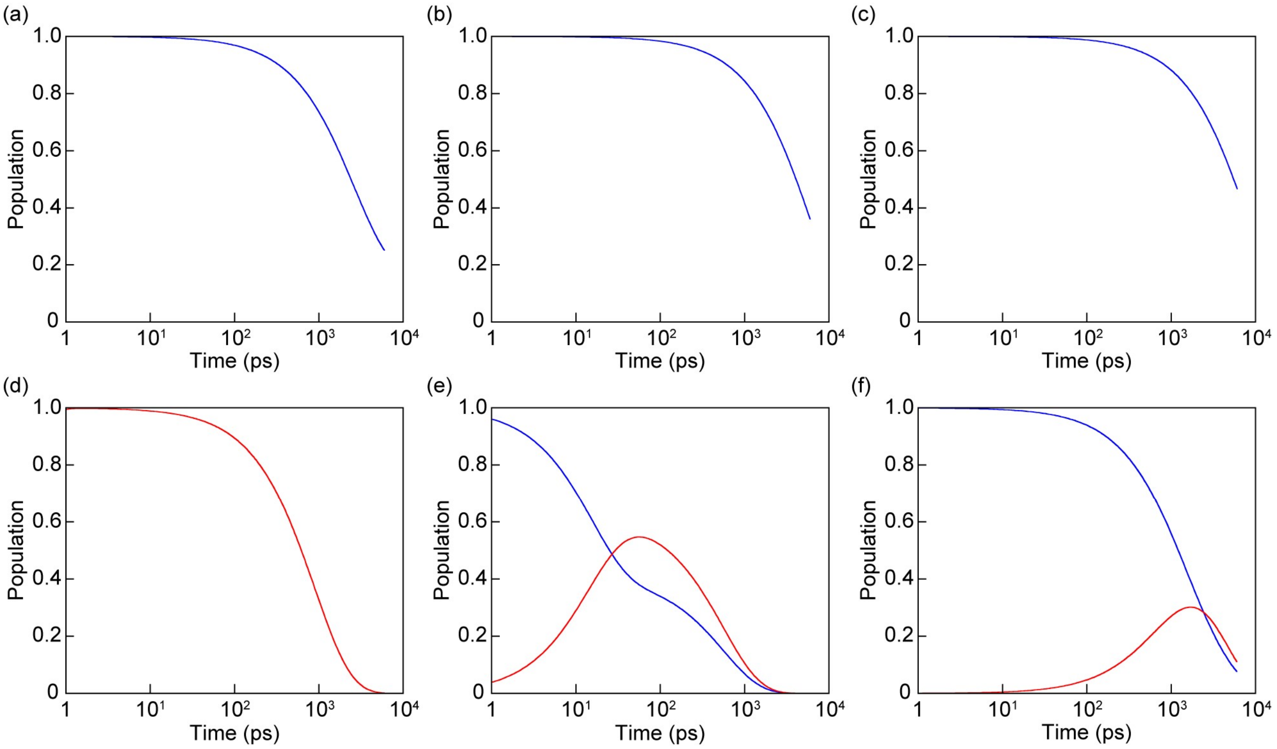

Fig. S7 Time-dependent population profiles of S_1 (blue line) and CT (red line) for (a) **R-An** (λ_{ex} = 480 nm), (b) **R-Te** (λ_{ex} = 580 nm), (c) **R-Pe** (λ_{ex} = 670 nm), (d) **Rot-An** (λ_{ex} = 480 nm), (e) **Rot-Te** (λ_{ex} = 580 nm), and (f) **Rot-Pe** (λ_{ex} = 680 nm) in toluene.

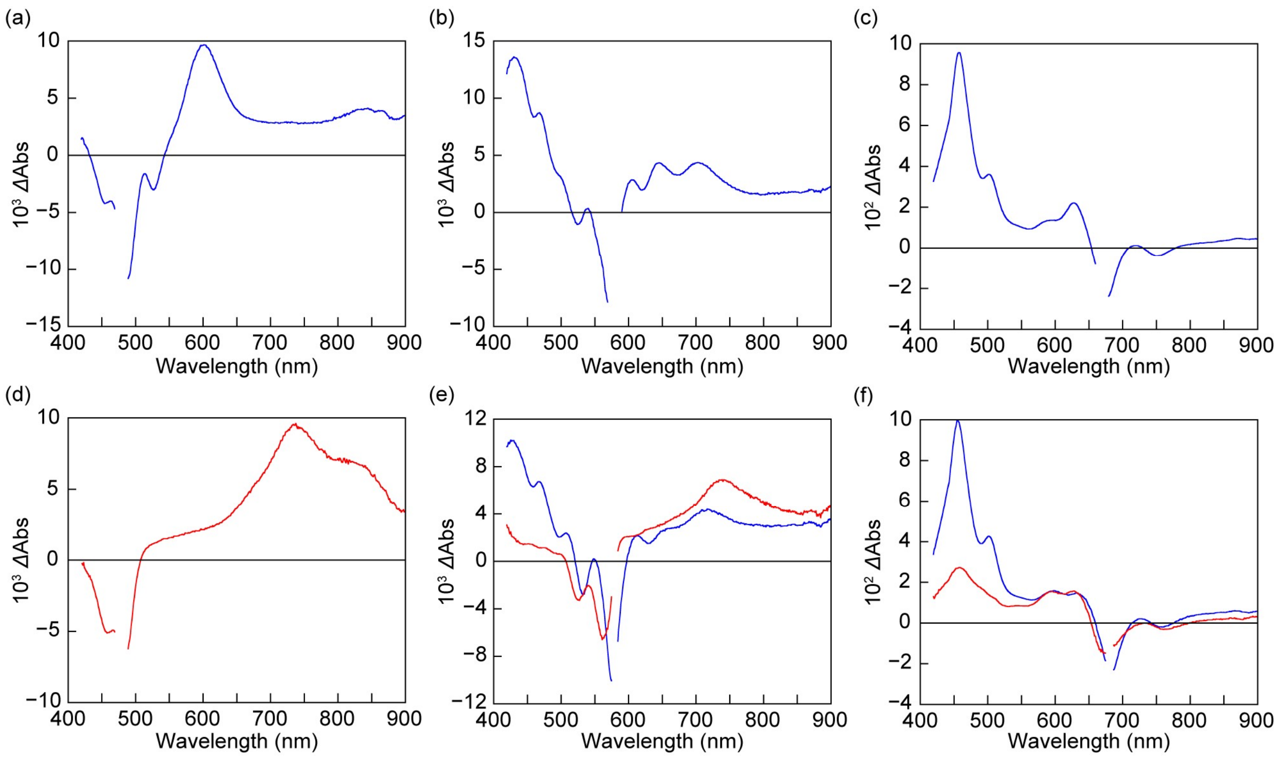

Fig. S8 Species associated spectra (SAS) of (a) **R-An** (λ_{ex} = 480 nm), (b) **R-Te** (λ_{ex} = 580 nm), (c) **R-Pe** (λ_{ex} = 670 nm), (d) **Rot-An** (λ_{ex} = 480 nm), (e) **Rot-Te** (λ_{ex} = 580 nm), and (f) **Rot-Pe** (λ_{ex} = 680 nm) in toluene. The blue lines represent the S_1 state, and the red lines represent the CT state.

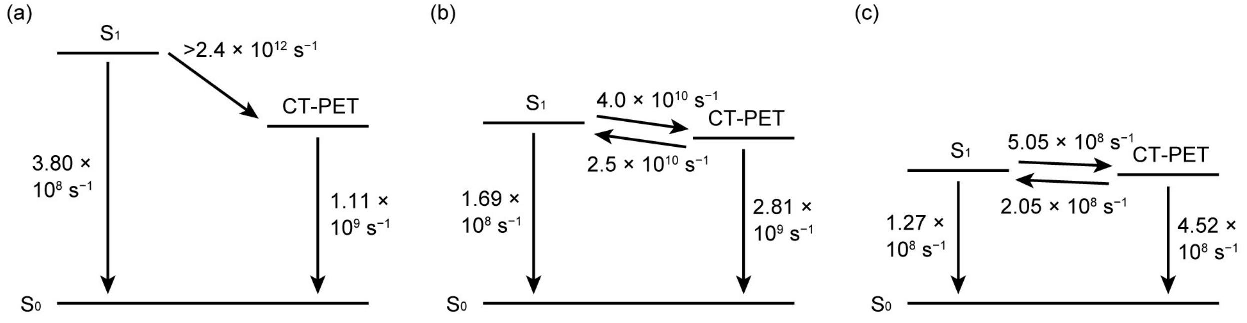

Fig. S9 Kinetic scheme of (a) **Rot-An**, (b) **Rot-Te**, and (c) **Rot-Pe** in toluene.

Theoretical Calculations

HOMO-LUMO energy level calculations

All quantum chemical calculations were performed using the GAUSSIAN 16 revision C01 program. Geometry optimizations and HOMO–LUMO energy level calculations were carried out using density functional theory (DFT) with the B3LYP functional and the 6-311G(d,p) basis set. Solvent effects were considered by applying the SMD implicit solvation model using toluene as the solvent. The rotaxanes studied in this work are composed of a quencher-containing axle molecule and fluorophore-containing cyclic molecules. Both components are functionalized with oligoethylene glycol side chains to ensure solubility. To reduce computational cost without significantly affecting the π-conjugated system, the oligoethylene glycol chains were simplified: they were replaced with methyl groups in the quencher axle molecule (**TC-PMDI**), and with methoxy groups in the fluorophore cyclic molecules (**TC-An**, **TC-Te**, and **TC-Pe**). The structures of these simplified model compounds are shown in Fig. S10.

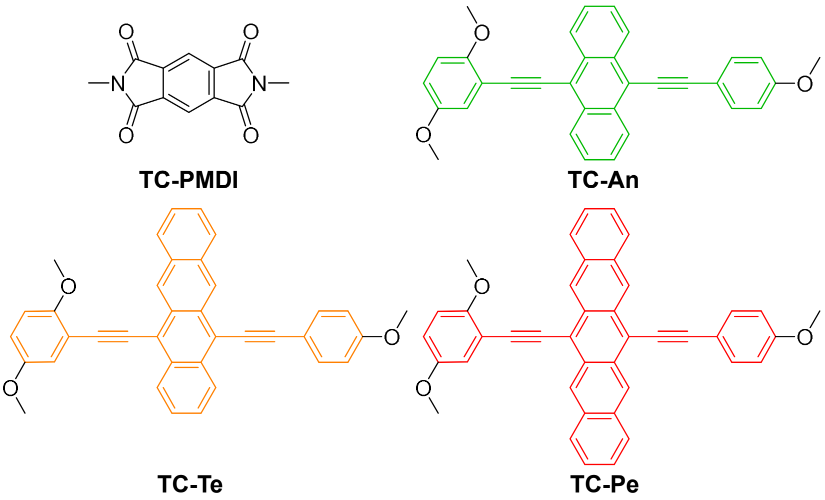

Fig. S10 Molecular structures of simplified model compounds **TC-PMDI**, **TC-An**, **TC-Te**, and **TC-Pe**. Side chains were replaced with methyl or methoxy groups to reduce computational complexity without significantly altering the π-conjugated electronic structure.

These calculations were used to estimate the HOMO and LUMO energy levels of each fluorophore and quencher. In particular, the energy gap between the fluorophore LUMO and quencher LUMO was examined to qualitatively assess differences in PET efficiency among acene fluorophores. Fig. S11 shows the HOMO and LUMO energy levels and orbital distributions obtained from DFT calculations for the simplified structures. The LUMO level of **TC-PMDI** lies lower than those of all fluorophores, suggesting that PET from the excited fluorophores to the quencher is energetically favorable. The variation in LUMO levels among the fluorophores are consistent with the observed quenching behavior.

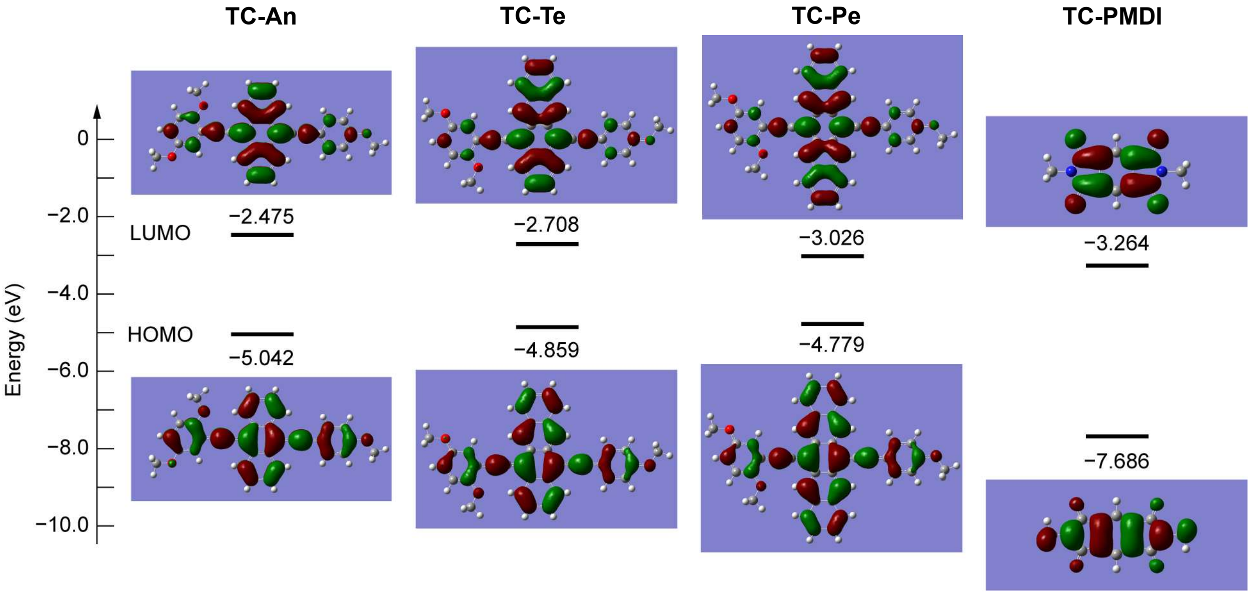

Fig. S11 DFT (B3LYP/6-311G(d,p), SMD=toluene) calculated HOMO and LUMO and their energy levels (in eV) for simplified model compounds.

^{1}H NMR Spectra of Polyurethanes

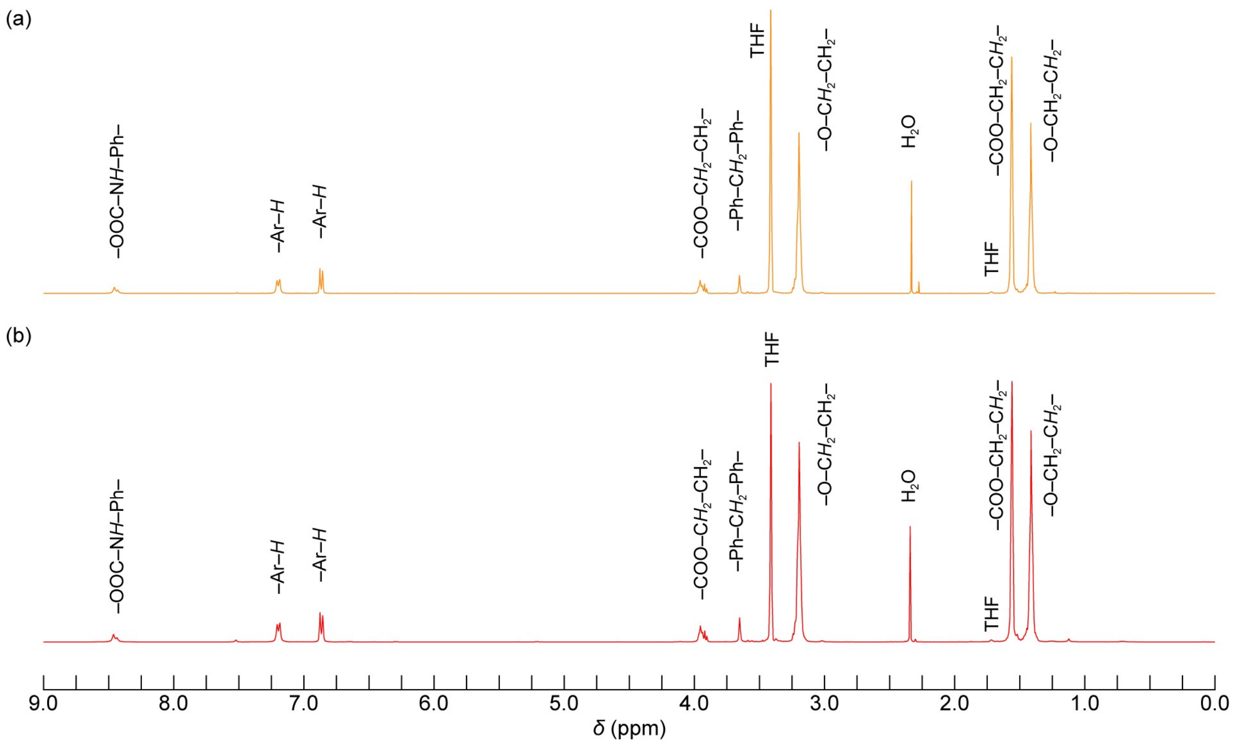

Fig. S12 ^{1}H NMR spectra of (a) **Rot-Te-PU** and (b) **Rot-Pe-PU**. All spectra were measured in THF-d_8 at 293 K.

Absorption and Photoluminescence Spectra of Polyurethanes in Solutions

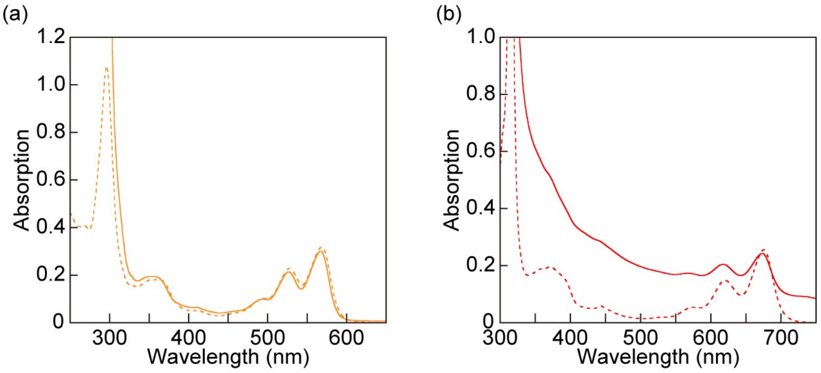

Fig. S13 (a) Absorption spectra of **Rot-Te** ($c = 1.0 \times 10^{-5}$ M, yellow dotted line) in $CHCl_3$ and **Rot-Te-PU** (yellow solid line) in THF. (b) Absorption spectra of **Rot-Pe** ($c = 1.0 \times 10^{-5}$ M, red dotted line) in $CHCl_3$ and **Rot-Pe-PU** (red solid line) in THF. The concentrations of the **Rot-Te-PU** and **Rot-Pe-PU** solutions were adjusted so that the absorbances at 567 and 674 nm match those of the solutions of **Rot-Te** and **Rot-Pe**, respectively.

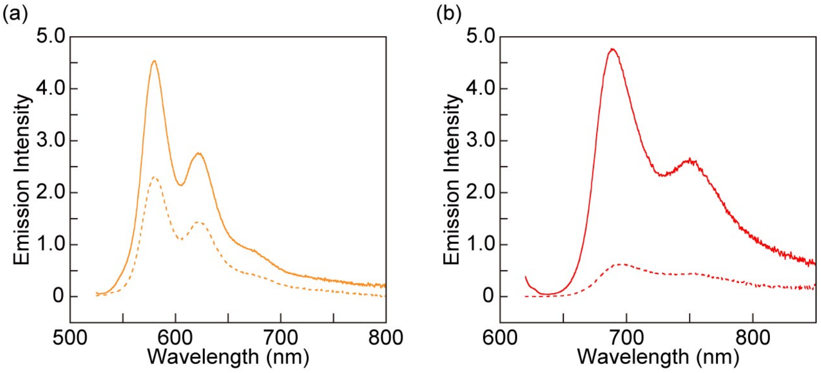

Fig. S14 (a) Fluorescence spectra of **Rot-Te** ($c = 1.0 \times 10^{-5}$ M, yellow dotted line) in $CHCl_3$ and **Rot-Te-PU** (yellow solid line) in THF. $\lambda_{ex} = 520$ nm. (b) Fluorescence spectra of **Rot-Pe** ($c = 1.0 \times 10^{-5}$ M, red dotted line) in $CHCl_3$ and **Rot-Pe-PU** (red solid line) in THF. $\lambda_{ex} = 615$ nm. The **Rot-Te-PU** and **Rot-Pe-PU** solutions were the same solutions as used in Fig. S13.

Thermal Properties of Polyurethanes

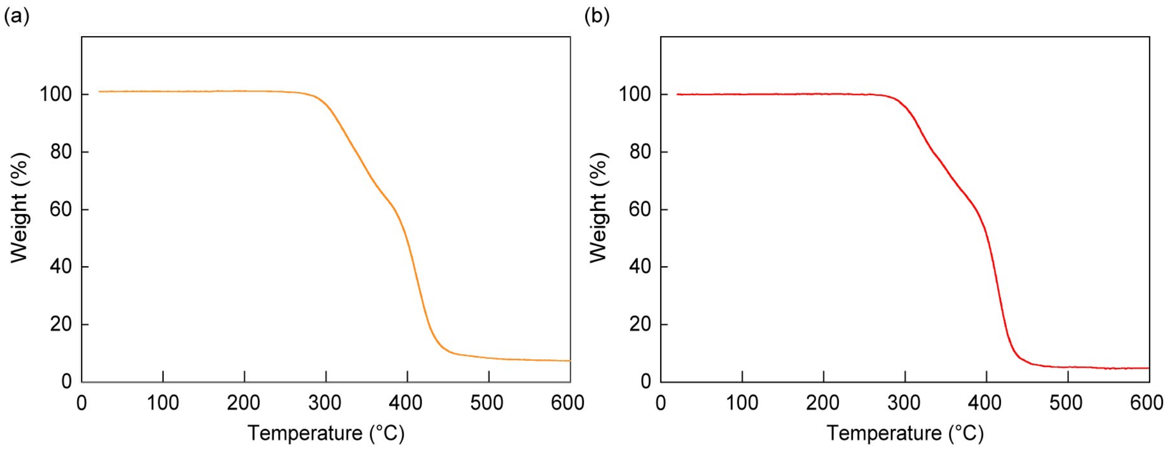

Fig. S15 TGA traces of (a) **Rot-Te-PU** and (b) **Rot-Pe-PU**. The heating rate was 10 °C/min.

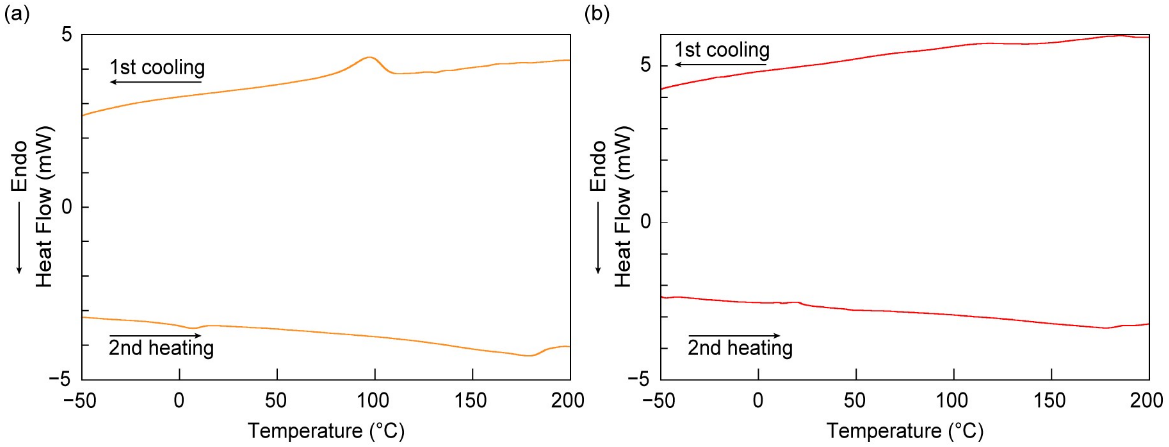

Fig. S16 DSC traces of (a) **Rot-Te-PU** and (b) **Rot-Pe-PU**. The heating and cooling rates were 10 °C/min.

Mechanical Properties of Polyurethanes

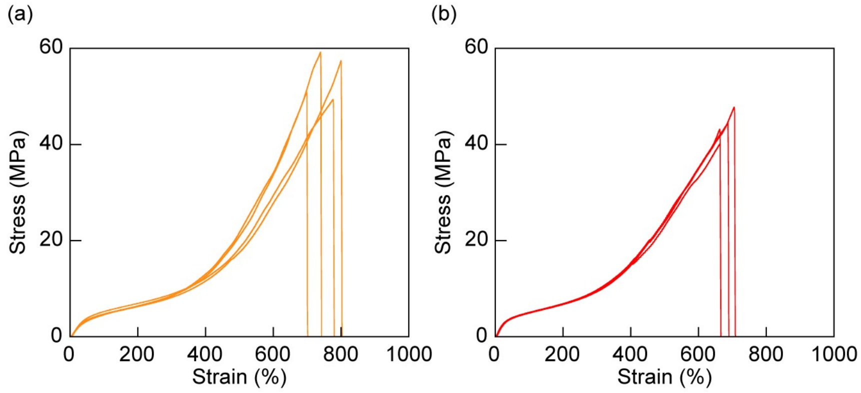

Fig. S17 Stress-strain curves of (a) **Rot-Te-PU** and (b) **Rot-Pe-PU** films. Each graph displays data obtained from the four individual specimens. The tests were performed with a strain rate of 300 mm/min at r.t.

Table S4. Overview of the mechanical properties of **Rot-Te-PU** and **Rot-Pe-PU** films as determined from stress-strain curves recorded during uniaxial tensile deformation (see Fig. S17).[a)]

	Elongation at break (%)	Stress at break (MPa)	Young's modulus (MPa)[b)]
**Rot-Te-PU**	754 ± 44	54 ± 5	11.0 ± 0.8
**Rot-Pe-PU**	754 ± 21	44 ± 3	13.9 ± 0.3

[a)] All data were extracted from the stress-strain curves shown in Fig. S17 and represent averages of 4 measurements ± standard deviation. [b)] The Young's moduli were derived from the slopes of the stress-strain curves in the strain regime between 6–16%.

NMR Spectra

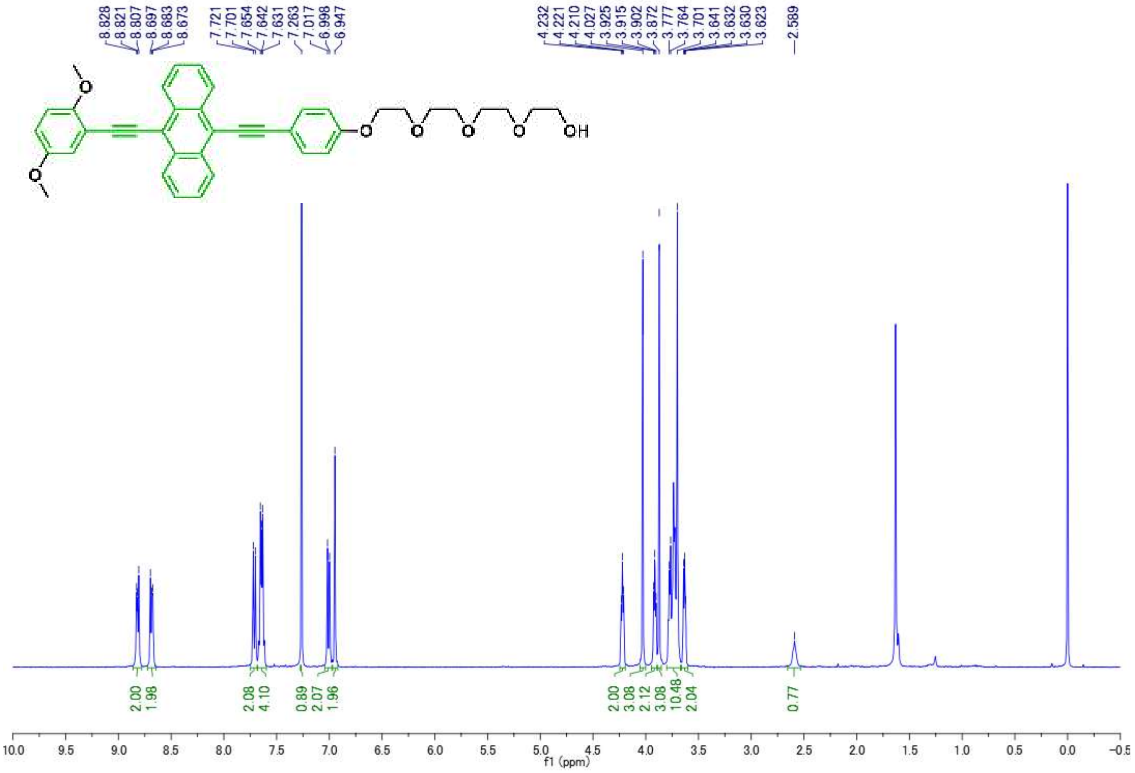

^{1}H NMR (400 MHz, $CDCl_3$) spectrum of **R-An**.

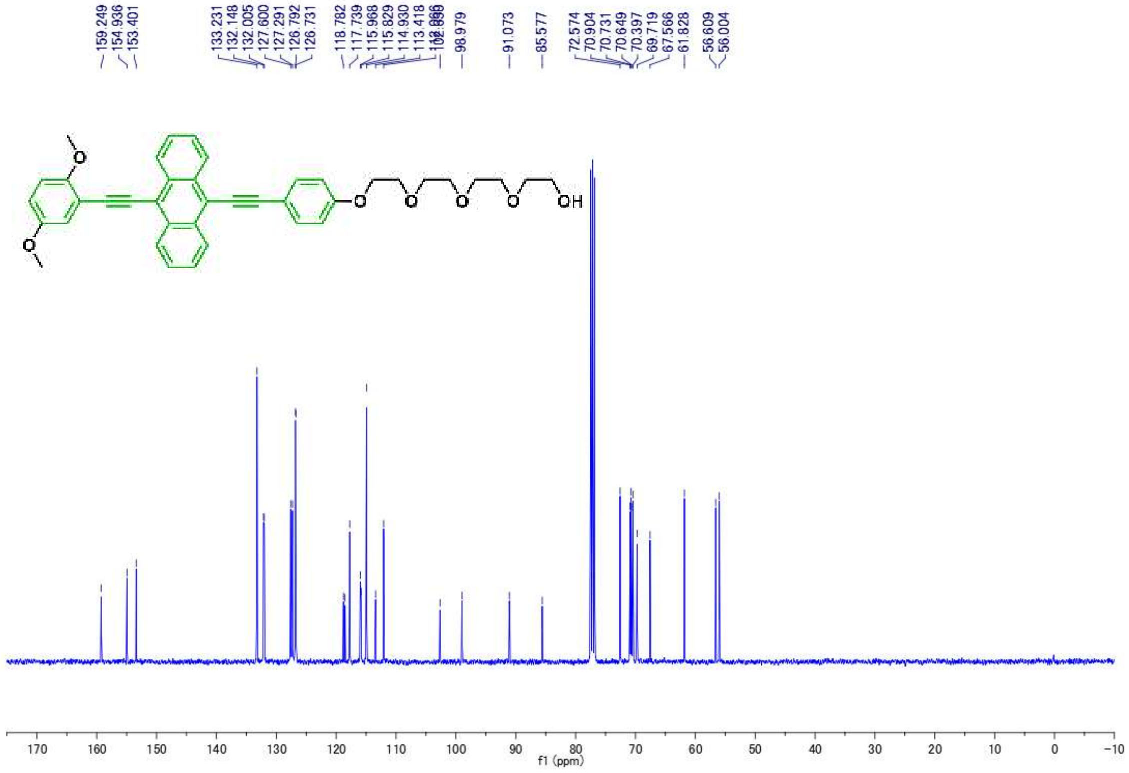

^{13}C NMR (100 MHz, $CDCl_3$) spectrum of **R-An**

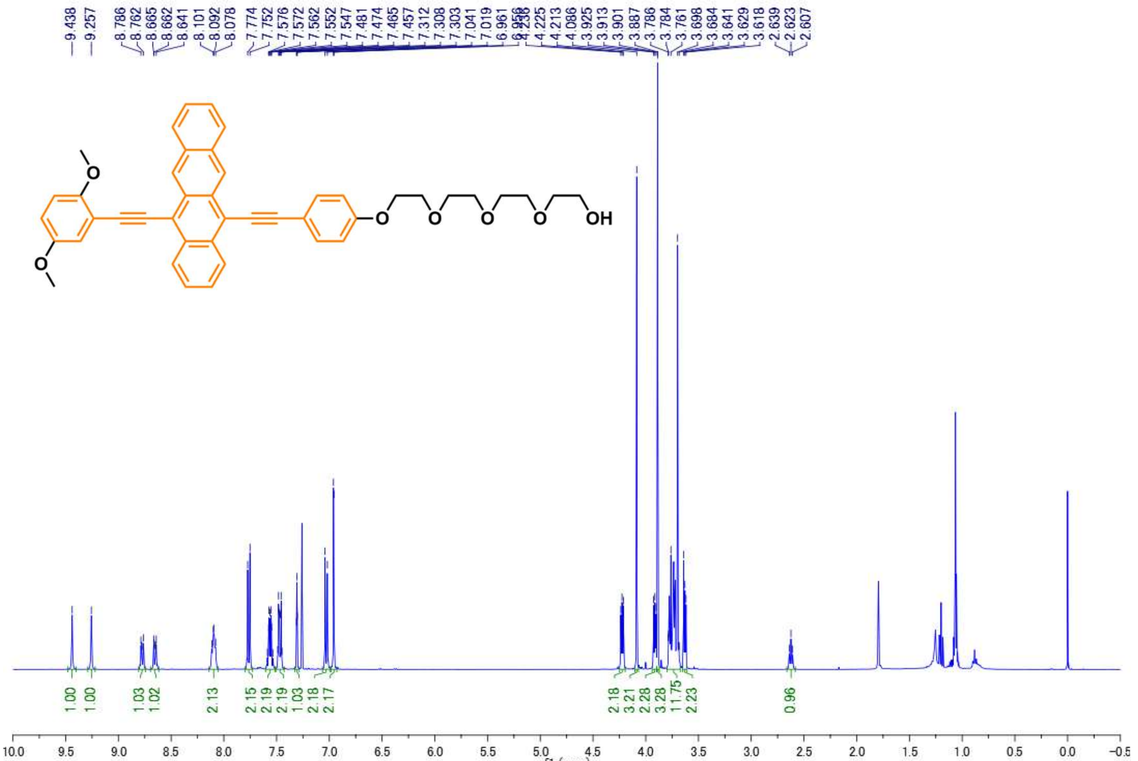

^{1}H NMR (400 MHz, $CDCl_3$) spectrum of **R-Te**

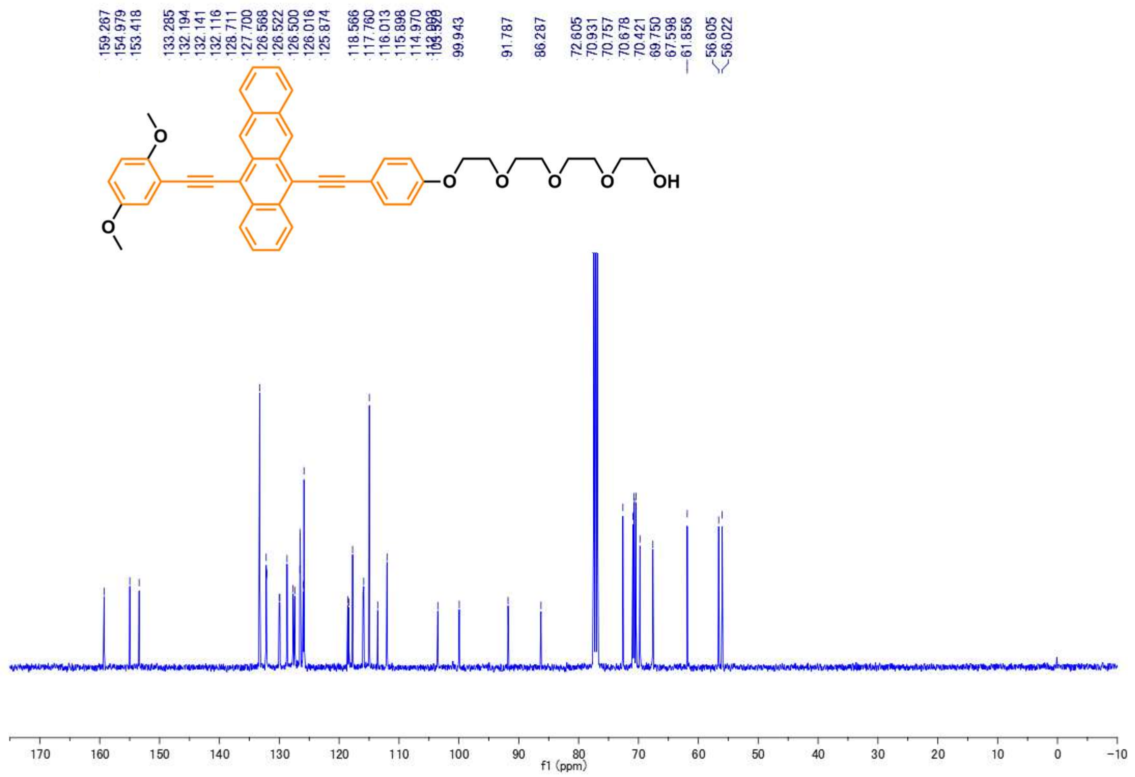

^{13}C NMR (100 MHz, $CDCl_3$) spectrum of **R-Te**

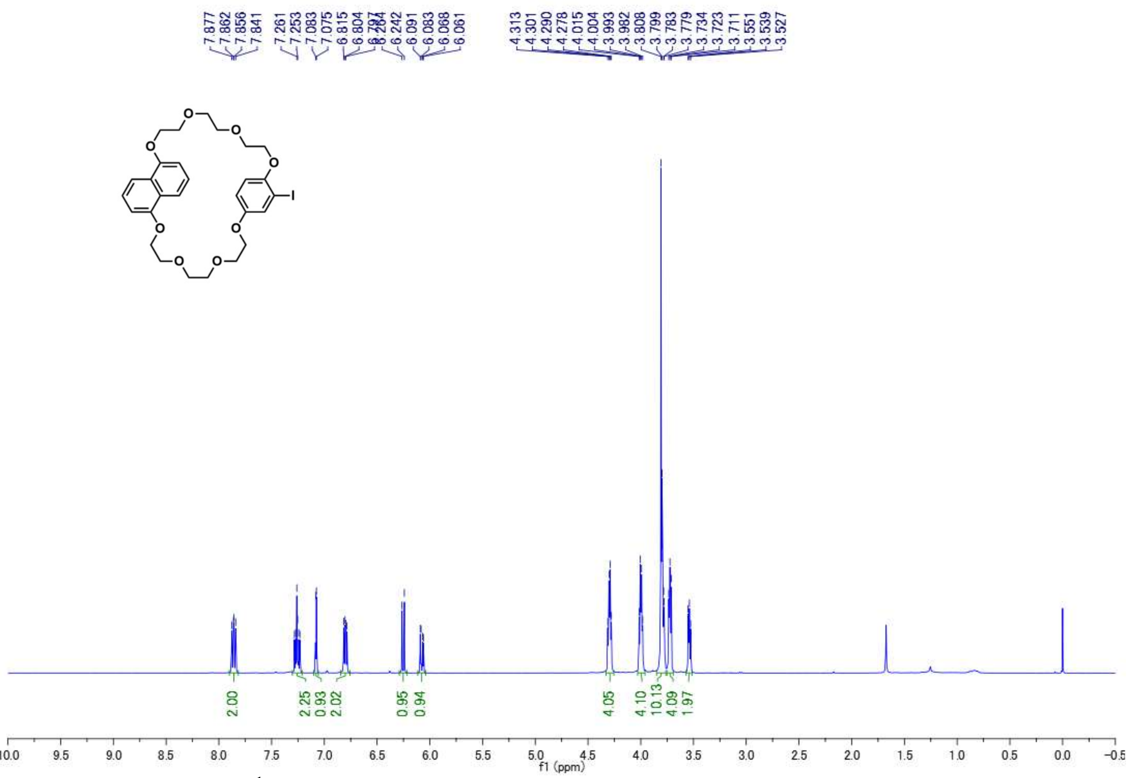

^{1}H NMR (400 MHz, $CDCl_3$) spectrum of **compound 1**

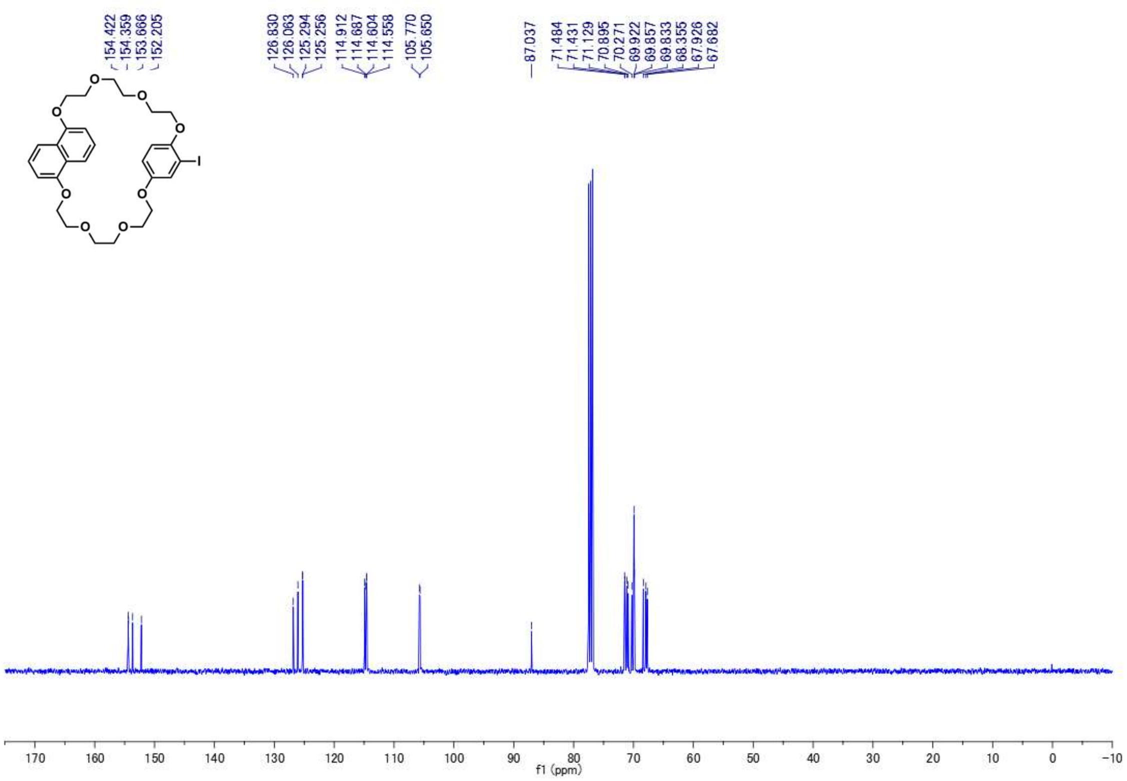

^{13}C NMR (100 MHz, $CDCl_3$) spectrum of **compound 1**

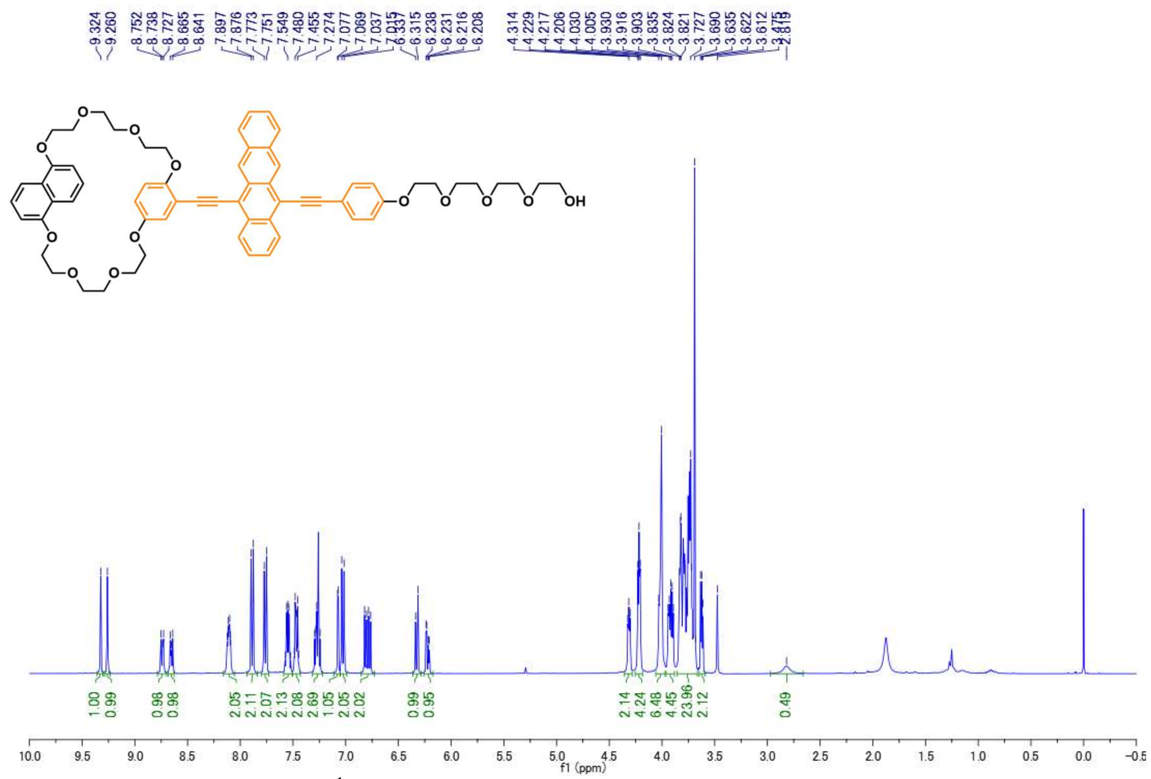

^{1}H NMR (400 MHz, $CDCl_3$) spectrum of **Te**

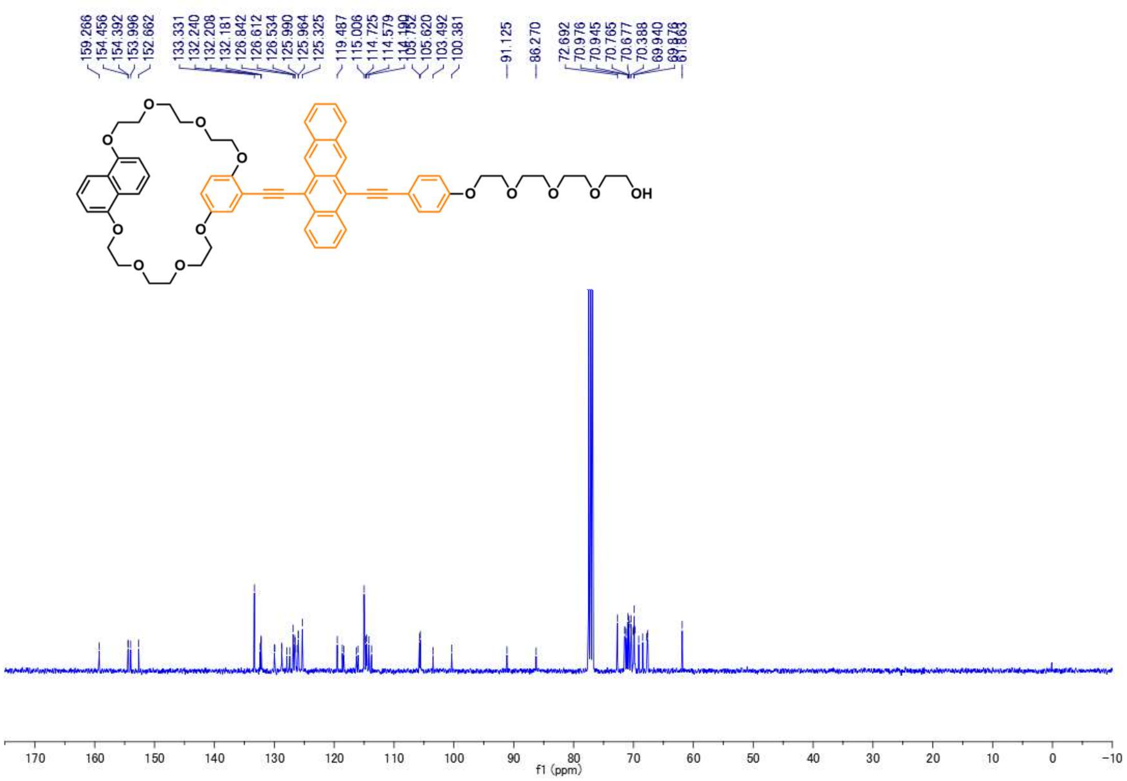

^{13}C NMR (100 MHz, $CDCl_3$) spectrum of **Te**

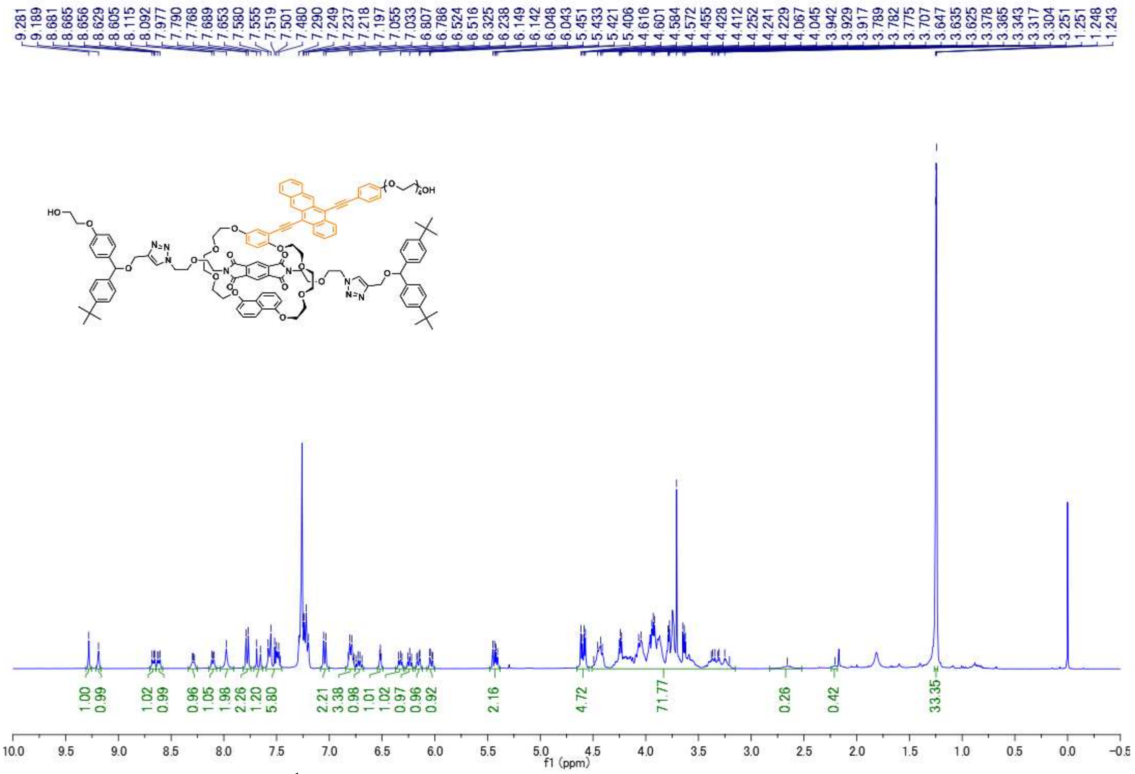

^{1}H NMR (400 MHz, $CDCl_3$) spectrum of **Rot-Te**

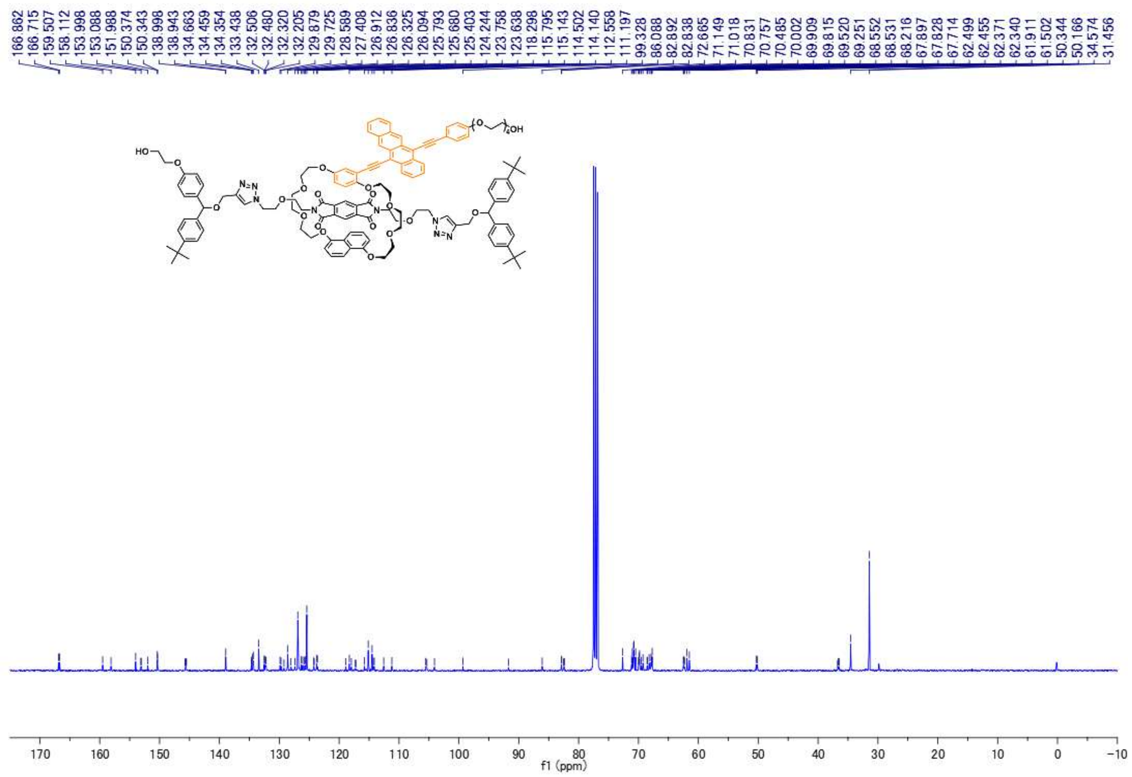

^{13}C NMR (100 MHz, $CDCl_3$) spectrum of **Rot-Te**

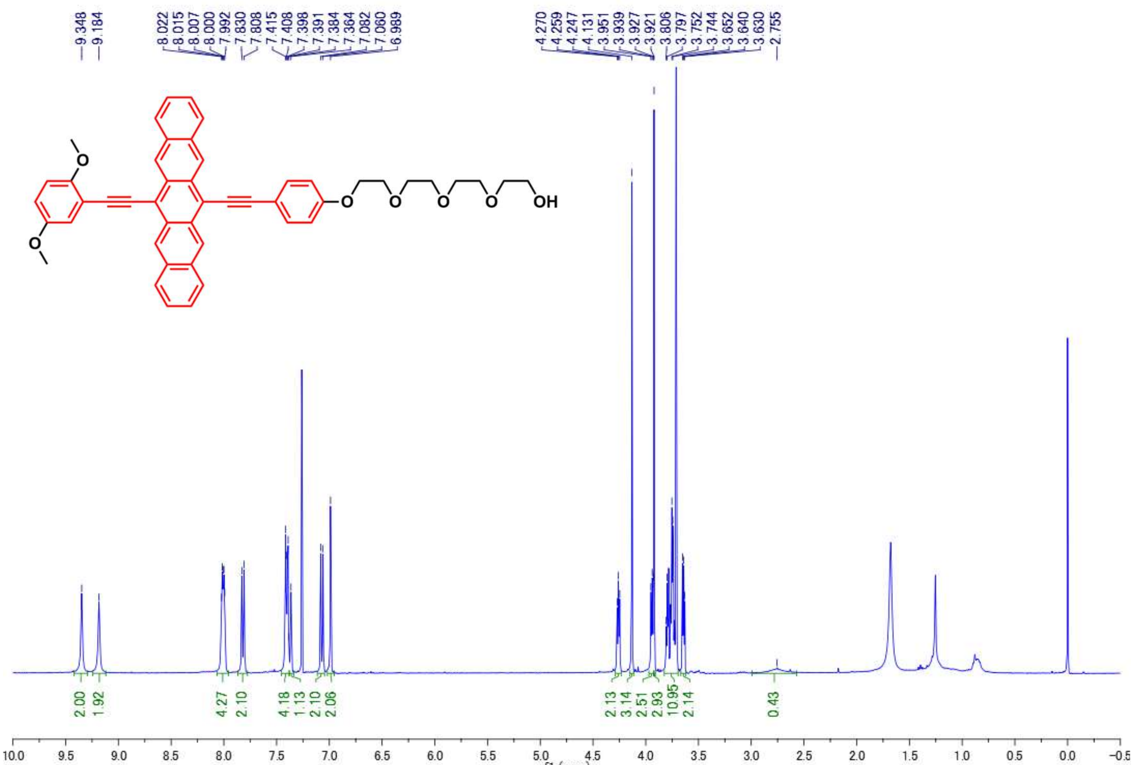

^{1}H NMR (400 MHz, $CDCl_3$) spectrum of **R-Pe**

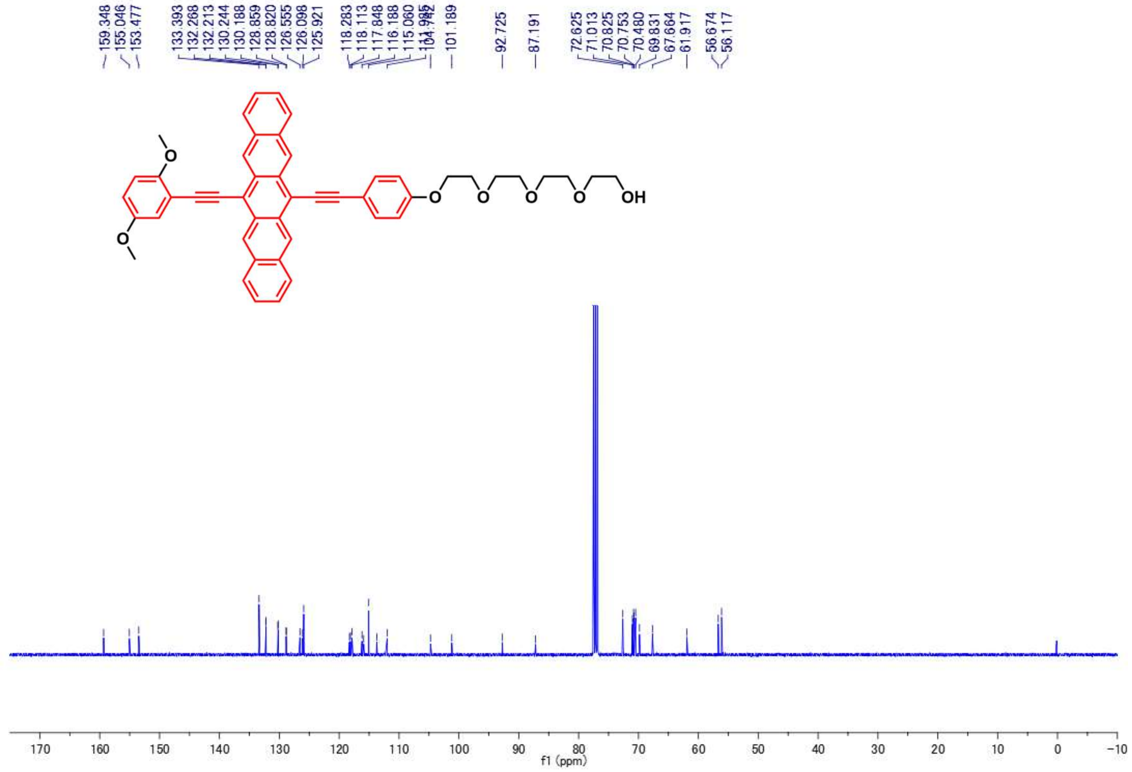

^{13}C NMR (100 MHz, $CDCl_3$) spectrum of **R-Pe**

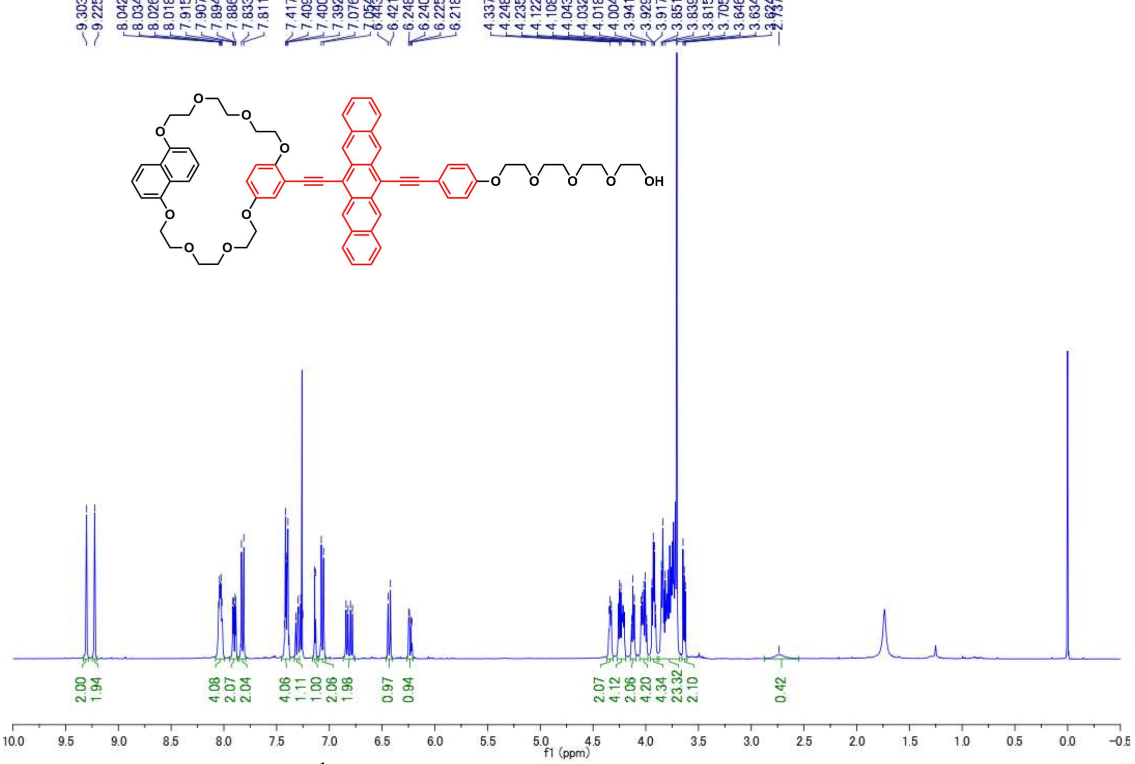

^{1}H NMR (400 MHz, $CDCl_3$) spectrum of **Pe**

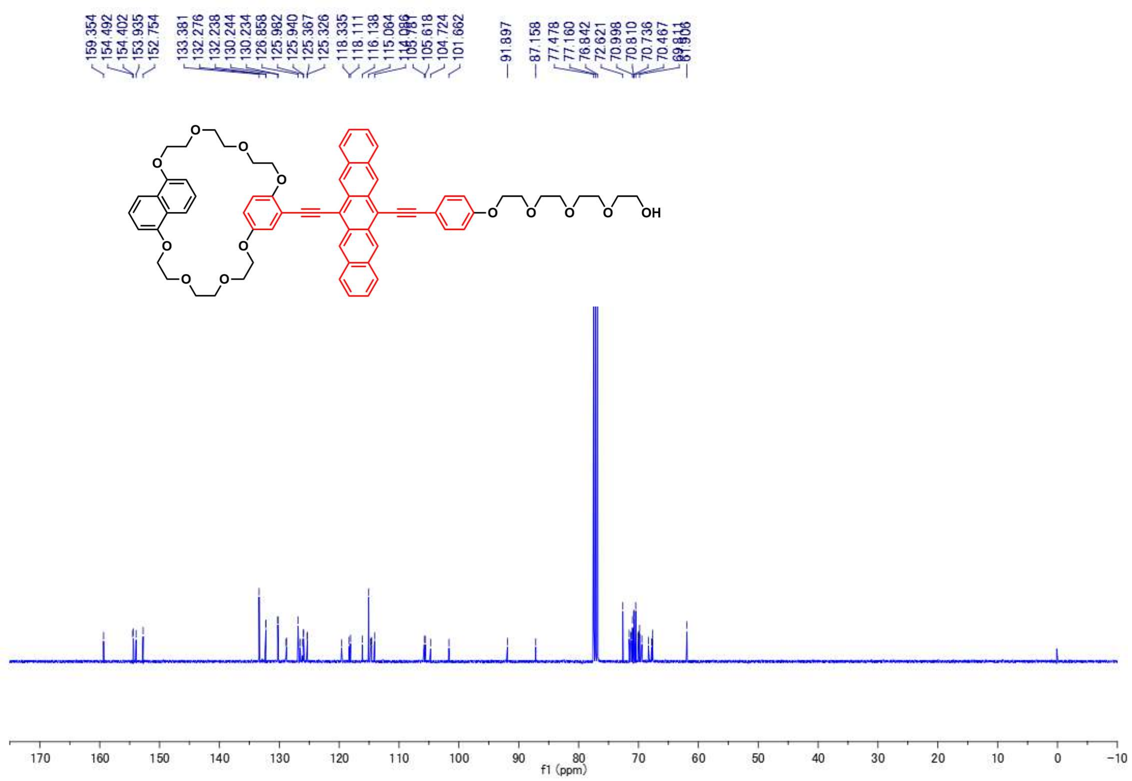

^{13}C NMR (100 MHz, $CDCl_3$) spectrum of **Pe**

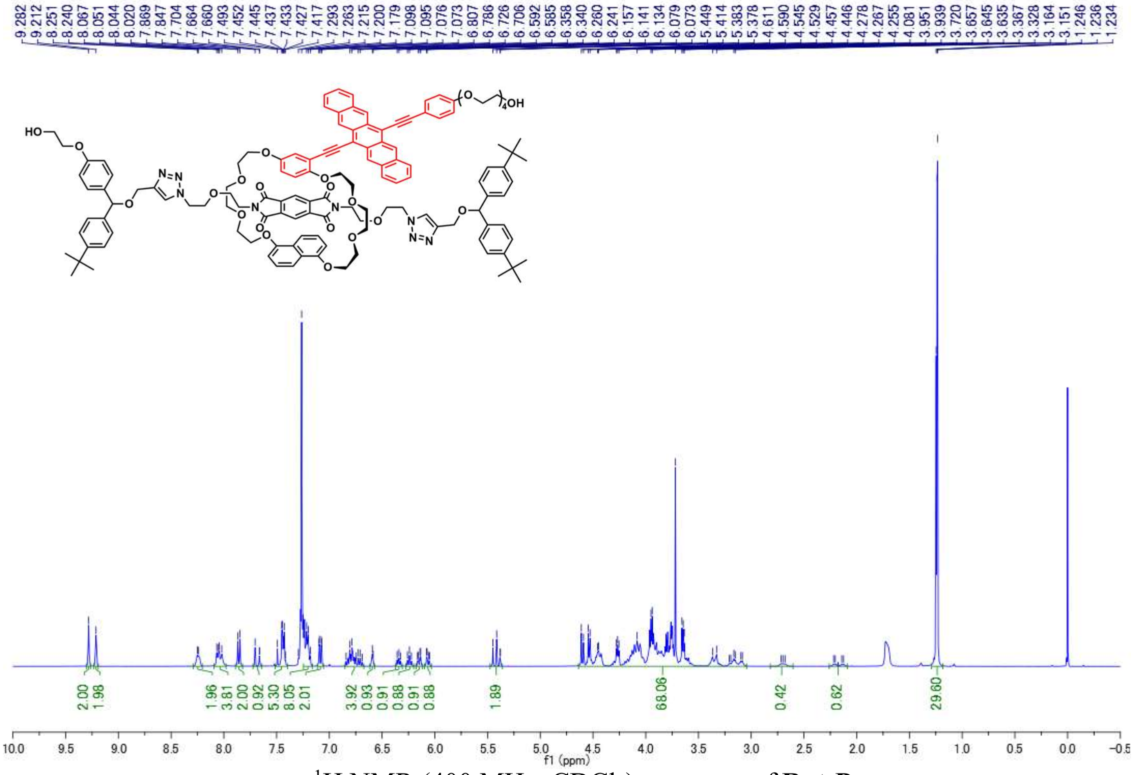

^{1}H NMR (400 MHz, $CDCl_3$) spectrum of **Rot-Pe**

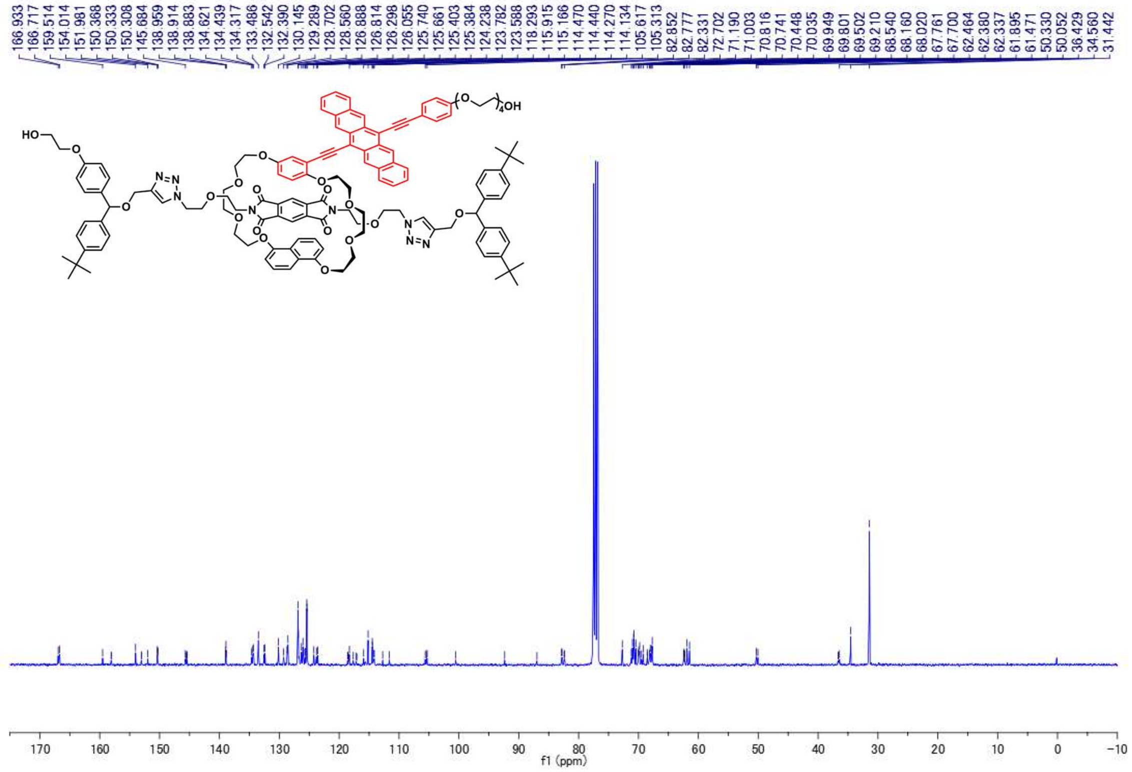

^{13}C NMR (100 MHz, $CDCl_3$) spectrum of **Rot-Pe**

References

S1. J. J. Snellenburg, S. Laptenok, R. Seger, K. M. Mullen and I. H. M. van Stokkum, *J. Stat. Softw.*, 2012, **49**, 1–22.

S2. R. C. Larock and L. W. Harrison, *J. Am. Chem. Soc.*, 1984, **106**, 4218–4227.

S3. Y.-P. Ou, C. Jiang, D. Wu, J. Xia, J. Yin, S. Jin, G.-A. Yu and S. H. Liu, *Organometallics*, 2011, **30**, 5763–5770.

S4. J. H. Hunter, L. Prendergast, L. F. Valente, A. Madin, G. Pairaudeau and M. J. Waring, *Bioconjug. Chem.*, 2020, **31**, 149–155.

S5. J. Zhang, S. Sarrafpour, T. E. Haas, P. Müller and S. W. Thomas, *J. Mater. Chem.*, 2012, **22**, 6182–6189.

S6. T. Muramatsu, Y. Okado, H. Traeger, S. Schrettl, N. Tamaoki, C. Weder and Y. Sagara, *J. Am. Chem. Soc.*, 2021, **143**, 9884–9892.

S7. W. Fang, J. Zhang, M. Guo, Y. Zhao and A. C.-H. Sue, *Angew. Chem., Int. Ed.*, 2024, **63**, e202409120.

S8. A. Yamagishi, Y. Egoshi, M. T. Fujiwara, N. Suzuki, T. Taniguchi, R. D. Itoh, Y. Suzuki, Y. Masuyama, K. Monde and T. Usuki, *Chem. Eur. J.*, 2023, **29**, e202203396.

S9. R. Mori, C. Weder and Y. Sagara, *Macromolecules*, 2023, **56**, 9248–9254.

S10. K. Hiratsuka, T. Muramatsu, T. Seki, C. Weder, G. Watanabe and Y. Sagara, *J. Mater. Chem. C*, 2023, **11**, 3949–3955.

S11. D. Lehnherr, A. H. Murray, R. McDonald and R. R. Tykwinski, *Angew. Chem., Int. Ed.*, 2010, **49**, 6190–6194.

S12. H. Imahori, K. Hagiwara, M. Aoki, T. Akiyama, S. Taniguchi, T. Okada, M. Shirakawa and Y. Sakata, *J. Am. Chem. Soc.*, 1996, **118**, 11771–11782.

S13. X. Wang, L. Lv, T. Li, C. Chen, X. Fan, B. Cui, L. Tang, Y. Chen, H. Liu and X. Li, *Chem. Eur. J.*, 2025, **31**, e202403125.

S14. X. Chen, A. A. Sukhanov, Y. Yan, D. Bese, C. Bese, J. Zhao, V. K. Voronkova, A. Barbon and H. G. Yaglioglu, *Angew. Chem., Int. Ed.*, 2022, **61**, e202203758.

S15. M.-A. Tehfe, J. Lalevée, F. Morlet-Savary, B. Graff, N. Blanchard and J.-P. Fouassier, *Macromolecules*, 2012, **45**, 1746–1752.

S16. Y. Hu, M. B. Thomas, W. A. Webre, A. Moss, R. G. W. Jinadasa, V. N. Nesterov, F. D'Souza and H. Wang, *Angew. Chem., Int. Ed.*, 2020, **59**, 20075–20082.

S17. M. Montalti, A. Credi, L. Prodi and M. T. Gandolfi, *Handbook of Photochemistry*, 3rd edn, CRC Press, Boca Raton, FL, 2006.
